# Efficient propyne/propadiene separation by microporous crystalline physiadsorbents

**DOI:** 10.1038/s41467-021-25980-y

**Published:** 2021-10-01

**Authors:** Yun-Lei Peng, Ting Wang, Chaonan Jin, Cheng-Hua Deng, Yanming Zhao, Wansheng Liu, Katherine A. Forrest, Rajamani Krishna, Yao Chen, Tony Pham, Brian Space, Peng Cheng, Michael J. Zaworotko, Zhenjie Zhang

**Affiliations:** 1 College of Chemistry, Nankai University, Tianjin, 300071 People’s Republic of China; 2 Department of Chemical Sciences, Bernal Institute, University of Limerick, Limerick, V94 T9PX Republic of Ireland; 3 State Key Laboratory of Medicinal Chemical Biology, Nankai University, Tianjin, 300071 People’s Republic of China; 4 Henan Institute of Advanced Technology, Zhengzhou University, Zhengzhou, 450052 People’s Republic of China; 5 Department of Chemistry, University of South Florida; 4202 East Fowler Avenue, CHE205, Tampa, FL 33620-5250 USA; 6 Van’t Hoff Institute for Molecular Sciences, University of Amsterdam; Science Park 904, 1098 XH Amsterdam, The Netherlands; 7 Key Laboratory of Advanced Energy Materials Chemistry (MOE), Nankai University, Tianjin, 300071 People’s Republic of China; 8 Renewable Energy Conversion and Storage Center, Frontiers Science Center for New Organic Matter, Nankai University, Tianjin, 300071 People’s Republic of China

**Keywords:** Metal-organic frameworks, Metal-organic frameworks, Organic-inorganic nanostructures

## Abstract

Selective separation of propyne/propadiene mixture to obtain pure propadiene (allene), an essential feedstock for organic synthesis, remains an unsolved challenge in the petrochemical industry, thanks mainly to their similar physicochemical properties. We herein introduce a convenient and energy-efficient physisorptive approach to achieve propyne/propadiene separation using microporous metal-organic frameworks (MOFs). Specifically, HKUST-1, one of the most widely studied high surface area MOFs that is available commercially, is found to exhibit benchmark performance (propadiene production up to 69.6 cm^3^/g, purity > 99.5%) as verified by dynamic breakthrough experiments. Experimental and modeling studies provide insight into the performance of HKUST-1 and indicate that it can be attributed to a synergy between thermodynamics and kinetics that arises from abundant open metal sites and cage-based molecular traps in HKUST-1.

## Abstract (web summary)

The separation of propadiene from propyne/propadiene mixtures remains challenging. Here, the authors report a sorbent screening protocol and show that metal-organic frameworks (MOFs) with open metal sites and cage-based molecule traps exhibit high performance for propyne/propadiene separation.

## Introduction

**Fig. 1:**
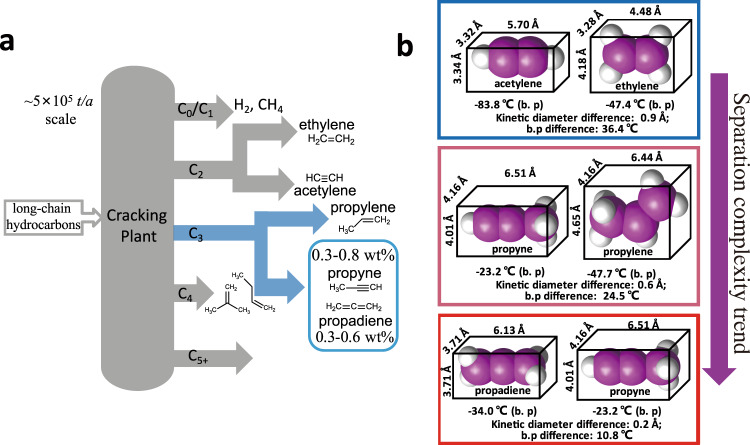
The major sources and separation complexity of light hydrocarbons. **a** Schematic diagram of the hydrocarbon cracking process. **b** Comparison of molecular size, kinetic diameter, and b.p difference of acetylene and ethylene, propyne and propylene and propyne and propadiene.

Propadiene (CH_2_ = C = CH_2_), the parent compound of allenes, is an important feedstock for organic synthesis. For instance, propadiene can serve as a chiral building block or as a useful reagent for cycloaddition reactions to form cyclobutane or trimethylenecyclohexane derivatives^1–5^. The combination of propadiene with inexpensive and environmentally benign hydrosilanes can serve as a replacement for stoichiometric quantities of allylmetal reagents, which are required in most enantioselective ketone allylation reactions^6^. Propadiene dimer (unsaturated four-membered ring hydrocarbons) prepared from prodadiene is also a useful and versatile starting material for valuable-added products^7^. At present, pure propadiene is produced via chemical synthesis with low yields, high energy consumption, and undesirable waste by-products^8–10^. A alternative approach to produce pure propadiene is urgently needed. Hydrocarbon cracking is among the largest-scale chemical processes in operation worldwide, converting over 500 million metric tons of feedstock per year to products such as α-olefins^11^. Propadiene is a by-product that constitutes 0.3–0.6 mass percent (wt%) of the total output and roughly 6 mole percent (mol%) of the crude C3 fraction (Fig. 1a)^12^. Propadiene is often available as a mixture with other gases such as propyne, from which it is difficult to separate due to their similar molecular sizes (difference < 0.2 Å) and close boiling points (difference = 10.8 °C) (Fig. 1b). Currently, there is still no effictive pathway to purify propadiene^13–16^, and propadiene-containing mixtures are generally processed as fuel to burn directly in industry which causes a lot of waste.

**Fig. 2:**
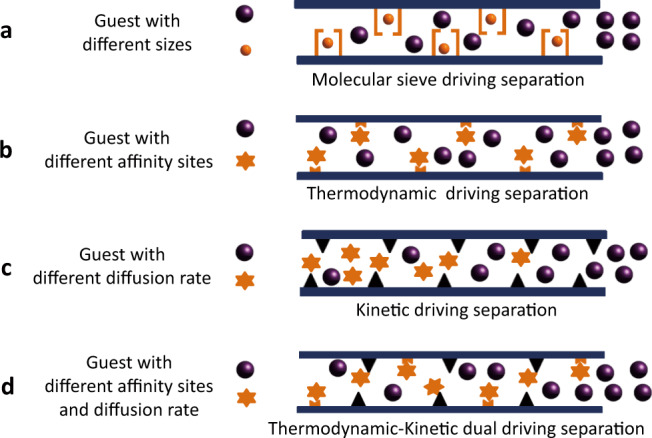
Illustration of adsorption separation mechanisms in physisorbents. **a** Molecular sieving. **b** Thermodynamically driven separation. **c** Kinetically driven mechanism. **d** Thermodynamic-kinetic dual-driving mechanism.

Physisorptive separation using porous sorbents has been of interest for some time^17–20^. In principle, sorbents can supercede traditional distillation separation methods and offer low energy consumption with easy operation and excellent safety. In the past two decades, metal-organic frameworks (MOFs), also known as porous coordination polymers, have emerged as a promising class of physisorbents for the capture, separation, and purification of light hydrocarbon gas mixtures^21,22^. Based on the concept of crystal engineering, MOFs can be fine-tuned in terms of pore size and pore chemistry^23–26^. in a manner that is infeasible for traditional porous sorbents such as activated carbon and zeolites^27,28^. The separation of gas mixtures via physisorption depends on several factors, including polarity, size, and shape of gas molecules. In general, the greater the difference in these factors, the easier it is to achieve efficient separation. The physisorptive separation of industrially gas mixtures such as ethylene/ethane^29–33^, ethylene/acetylene^19,34–36^, acetylene/carbon dioxide^37–39^, propylene/propyne^40,41^, and propane/propylene^20,42,43^ exemplify what seemed intractable challenges that have recently been addressed by microporous MOFs with suitable pore size and chemistry to enable selective binding. Propyne/propadiene separation, however, (Fig. 2a) remains unresolved. Whereas thermodynamically driven separation (Fig. 2b) has proven effective for other separations, it is mitigated here by the almost identical polarizability of propyne *vs*. propadiene (55.5 × 10^−25^ cm^3^ vs. 56.9 × 10^−25^ cm^3^)^44–46^. These critical requirements cause difficulty for separation propyne/propadiene mixtures^47^.

Here, we address the challenge of propyne/propadiene separation and report a thermodynamic-kinetic dual-drive separation strategy (Fig. 2d) using microporous MOF adsorbents with dense open metal sites (OMSs) and cage-based molecule traps which would offer the needed performance driven by two factors: (i) The C ≡ C moiety in propyne offers distinct binding to OMSs. (ii) The pore shapes of adsorbents can be adjusted to control gas diffusion to enhance the dynamic screening effect. Our study reveals that microporous MOFs with both a high-density of OMSs and restricted cage-based molecule traps, as exemplified by HKUST-1 and MOF-505, exhibit the best separation performance by comprehensive comparison of a range of candidate sorbent. Importantly, HKUST-1 is one of the few MOFs that has been manufactured at an industrial scale with current production methodology offering a space-time yield of 400,000 kg m^−3^ day^−1^ ^48^.

## Results and discussion

### Structure and basic characterizations

**Fig. 3:**
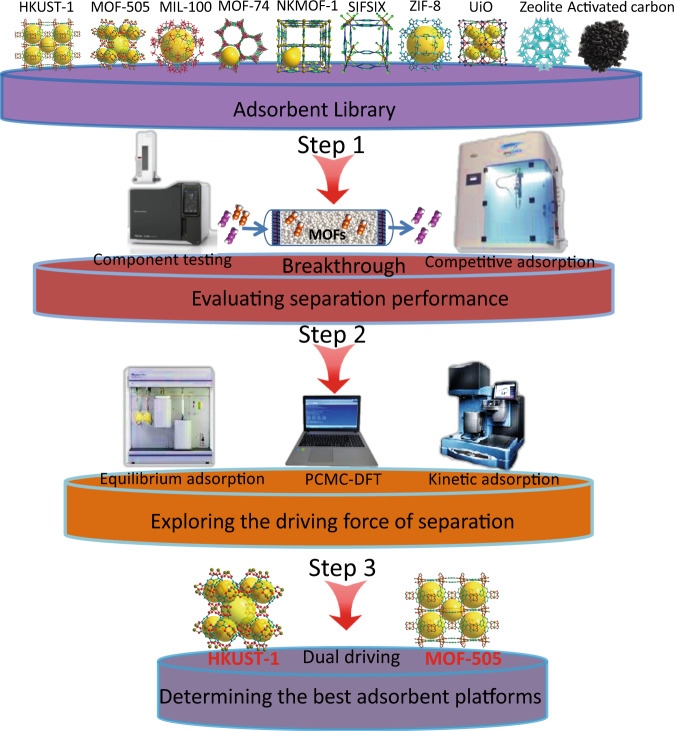
Illustration of the assay package used herein for determining propyne/propadiene separation performance.

We selected a sorbent library comprising traditional porous materials (activated carbon and zeolites) and three types of MOFs (14 different sorbents) classified as follows: (I) MOFs with OMSs, including HKUST-1, MOF-505, Mg-MOF-74, NKMOF-1-Ni, MIL-100-Cr, MIL-100-Fe and MIL-101-Cr; (II) Hybrid ultramicroporous MOFs with strong binding sites (e.g., SiF_6_^2−^ and NbOF_5_^2^^−^), including SIFSIX-2-Cu-i, SIFSIX-3-Ni, UTSA-200 (SIFSIX-14-Cu-i) and ZU-62; (III) MOFs without strong binding sites, including UiO-66, UiO-67, and ZIF-8 (Supplementary Table 1 for detailed structural parameters). Screening of this library was conducted by dynamic breakthrough tests to find the best performing candidates for propyne/propadiene separation. In addition, the equilibrium adsorption isotherms, simulated gas sorption studies, and kinetic adsorption behavior of the library were evaluated (Fig. 3). Activated carbon and zeolites were obtained via commercial sources. All MOFs samples in this study were prepared according to the previous procedures reported in the literature or using appropriately modified procedures. Powder X-ray diffraction (PXRD), BET surface area measurement, and scanning electron microscopy (SEM) data (Supplementary Figs. 1–6) confirmed that all MOFs possessed high crystallinity and the expected sorption parameters.

### The dynamic actual breakthrough experiment

**Fig. 4:**
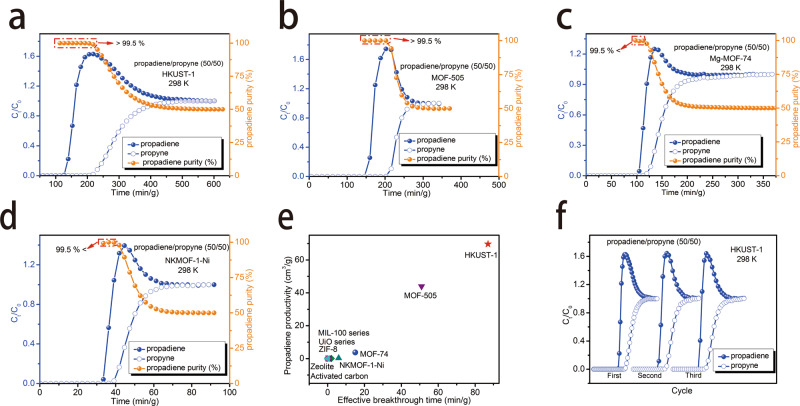
Experimental breakthrough curves of the MOFs with OMSs for propyne/propadiene (50/50, v/v) separation at 298 K. **a** HKUST-1. **b** MOF-505. **c** Mg-MOF-74. **d** NKMOF-1-Ni. **e** Comparing productivity of propadiene for corresponding breakthrough experiments in the adsorbent library. **f** Cycling tests of HKUST-1 for propyne/propadiene mixture (gas velocity: 2.0 mL/min).

Dynamic breakthrough experiments are the most direct and reliable way to evaluate sorption separation performance. Breakthrough experiments of propyne/propadiene mixtures that simulate vacuum swing adsorption processes were conducted on a custom-built separation apparatus in which propyne/propadiene (50/50, ν/ν) mixtures were used as feed gas (Supplementary Fig. 7). The dynamic breakthrough was performed using a packed column of the activated sorbent under a 2.0 mL/min flow of propyne/propadiene (50/50, ν/ν) at 298 K. Experimental results are presented in Fig. 4 and Supplementary Fig. 8. The breakthrough results revealed no separation for activated carbon and zeolites whereas some MOFs with OMSs were effective. As revealed by Fig. 4, propadiene was eluted through the adsorption bed while propyne was retained, affording >99.5% pure propadiene. The retention time of propyne after breakthrough was 87 min/g for HKUST-1 and 51 min/g for MOF-505. Although Mg-MOF-74 and NKMOF-1-Ni, other MOFs with a high-density of OMSs, also exhibited separation, retention times were only 15 and 6 min/g (propadiene purity > 99.5%, Fig. 4). Mesoporous MOFs with OMSs (MIL-100-Cr, MIL-100-Fe and MIL-101-Cr) showed no separation effect (Supplementary Fig. 8a–c). Type-II MOFs with strong functional sites, including SIFSIX-2-Cu-i, SIFSIX-3-Ni, UTSA-200, and ZU-62, produced propadiene with lower purity (<95%, Supplementary Fig. 8d–g). Type-III MOFs without strong binding sites (UiO-66, UiO-67, and ZIF-8) were ineffective (Supplementary Fig. 8h–j). Overall, the hierarchy of separation performance was as follows: type-I MOFs with OMSs > type-II MOFs with strong functional sites > type-III MOFs without strong binding sites. The hierarchy of retention time for MOFs with OMSs was determined to be HKUST-1 > MOF-505 > MOF-74 > NKMOF-1-Ni » MIL-100-Cr, MIL-100-Fe, and MIL-101-Cr. HKUST-1 and MOF-505 possessed the best separation performance (Fig. 4 and Supplementary Fig. 8). Propadiene production of HKUST-1 and MOF-505 from the outlet effluent for a given cycle was calculated to be 69.6 and 43.9 cm^3^/g, far exceeding that observed in Mg-MOF-74 (3.75 cm^3^/g) and other materials (Fig. 4e). HKUST-1 is the benchmark material for separating propyne/propadiens as it exhibited the highest productivity of pure propadiene (>99.5%) via a one-step separation process. In order to gain insight into the mechanism, we systematically explored the equilibrium and kinetic adsorption behavior of gas molecules through further experimental studies and modeling.

### Single-component equilibrium adsorption isotherms

**Fig. 5:**
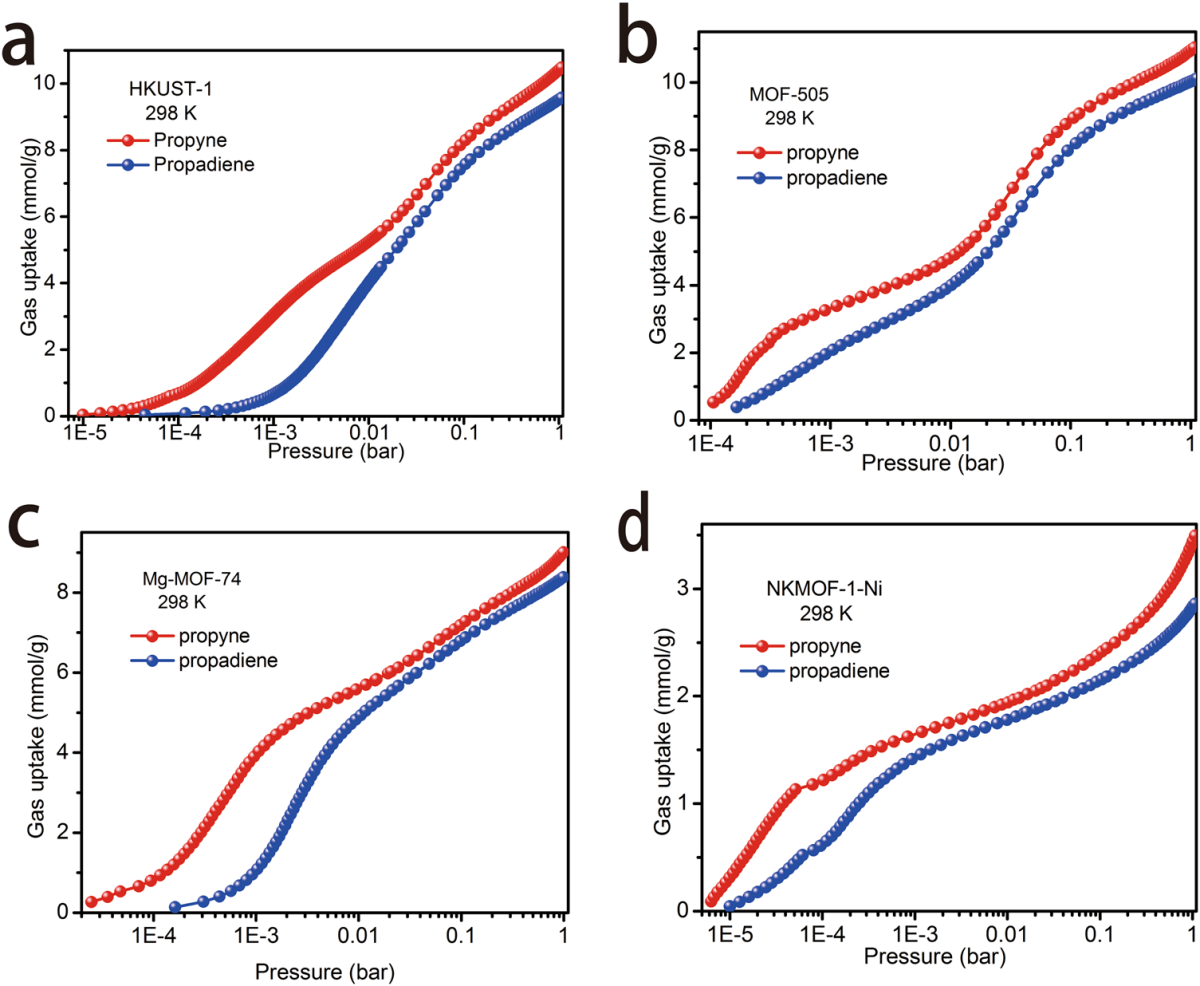
Propyne and propadiene equilibrium adsorption isotherms of the selected MOF materials at 298 K. **a** HKUST-1. **b** MOF-505. **c** Mg-MOF-74. **d** NKMOF-1-Ni.

Single-component equilibrium adsorption isotherms can directly show the adsorptive capacity of porous materials and provide indicators of sorbent-sorbate binding and separation selectivity. Propyne and propadiene equilibrium adsorption isotherms were measured at 273, 298, 308, and 318 K (Supplementary Figs. 9, 12–15). Type-III MOFs without strong binding sites possessed high adsorption capacity for both propyne and propadiene (Supplementary Fig. 9). However, they showed little difference in the adsorption profiles of propyne and propadiene over the pressure range tested. In the low-pressure area, the adsorption isotherms of propyne and propadiene overlapped, consistent with the dynamic breakthrough tests (Supplementary Fig. 8h–j). Type-II MOFs with electrostatic sites also showed similar adsorption behavior for propyne and propadiene, especially SIFSIX-3-Ni and UTSA-200 with isotherms overlapping from 0.1 to 1 bar, after reaching adsorption saturation at <0.1 bar. It was difficult to compare adsorption behavior at low pressure by a linear diagram (Supplementary Fig. 9) so we resorted to a logarithmic plot (Fig. 5 and Supplementary Fig. 10) which revealed similar sorption performance throughout the pressure range tested. Supplementary Fig. 9h, j reveal that SIFSIX-2-Cu-i and ZU-62 adsorb almost the same amount of propyne as propadiene, consistent with the relatively poor separation observed in our breakthrough experiments (Supplementary Fig. 8d and Supplementary Fig. 8f). Interestingly, although SIFSIX-3-Ni and UTSA-200 exhibited almost overlapping adsorption isotherms for propyne and propadiene from 0.1 to 1 bar, they exhibited preferential adsorption for propyne at 0.1 bar. However, they did not show strong separation performance in breakthrough experiments. As shown in Fig. 5, Type-I microporous MOFs with OMSs adsorbed propyne over propadiene throughout the pressure range tested, correlating with the breakthrough experiments detailed above (Fig. 4).

Isosteric heat of adsorption (*Q*_st_) values reflect the affinity of a sorbent for a sorbate and are an indicator of thermodynamically driven separation. Type-I microporous MOFs with OMSs could be classified into two categories: (i) HKUST-1 and MOF-505 with cage-based molecule trap structure (Supplementary Fig. 11); (ii) Mg-MOF-74 and NKMOF-1-Ni with the regular one-dimensional channel. Their *Q*_st_ were evaluated by the Clausius–Clapeyron equation through fitting the adsorption isotherm using dual-site Langmuir (DSL) model and dual-site Langmuir–Freundlich (DSLF) model equation (Supplementary Figs. 12–15). The initial *Q*_st_ values for propyne and propadiene for HKUST-1, MOF-505, Mg-MOF-74, and NKMOF-1-Ni were 42 and 40, 78 and 58, 53 and 46, 65 and 54 kJ mol^−1^, respectively (Supplementary Fig. 16). *Q*_st_ towards propyne was higher than that of propadiene for all optimized MOFs with OMSs. Massively Parallel MC (MPMC) theoretical calculations also indicated that the binding affinity for propyne was stronger than that for propadiene^47^.

**Fig. 6:**
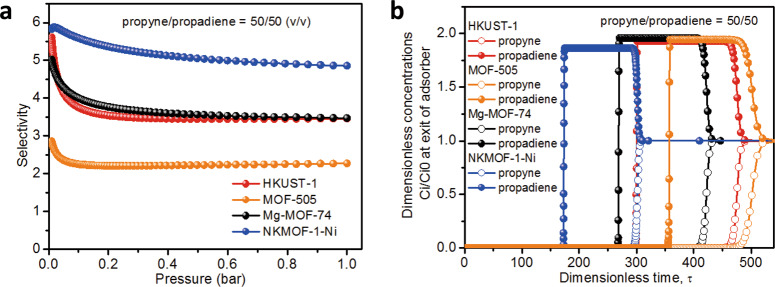
IAST adsorption selectivities and simulation breakthrough curves for separation of propyne/propadiene for 50/50 (ν/ν) at 298 K. **a** IAST adsorption selectivities. **b** Simulation breakthrough curves.

Ideal adsorbed solution theory (IAST) selectivity can be used to estimate thermodynamic separation performance. The selectivity of microporous MOFs with OMSs was calculated using IAST through fitting gas isotherm at 298 K by the DSL and DSLF equation (Supplementary Figs. 12–15). Considering the context of industrial separations, we calculated based on a propyne/propadiene (50/50, ν/ν) mixture at 298 K (Fig. 6a). NKMOF-1-Ni exhibited the highest selectivity of 6.0 with a selectivity > 4.8 across the pressure range. The selectivities of HKUST-1 and Mg-MOF-74 were slightly lower than NKMOF-1-Ni in the low-pressure area (<0.01 bar) and tended to be consistent with each other under the remaining pressure range (0.01–1 bar). These selectivity results indicate that microporous MOFs with OMSs materials should possess good separation performance.

In order to further evaluate separation performance, transient breakthrough simulations were conducted for propyne/propadiene feed mixtures (50/50, ν/ν) at 298 K and 1 bar using the methodology described in earlier publications^46^. Fig. 3b showed the outlet concentrations of propyne exiting in the fixed bed of HKUST-1, MOF-505, Mg-MOF-74, and NKMOF-1-Ni as a function of the dimensionless time, *τ*, for propyne/propadiene mixture. Although the IAST selectivity of NKMOF-1-Ni was the highest, the *τ* break value for NKMOF-1-Ni was not the highest, possibly attributing to its lowest uptake capacity for propyne. The simulated dynamic breakthrough results are related to adsorption selectivity, uptake capacity, and crystal density, etc. The hierarchy of the τ break values in the MOFs with OMSs is HKUST-1 > Mg-MOF-74 > MOF-505 > NKMOF-1-Ni. HKUST-1, MOF-505, Mg-MOF-74, and NKMOF-1-Ni demonstrated efficient and similar separation performance for propyne/propadiene (50/50, ν/ν) mixture.

### Molecular simulation

**Fig. 7:**
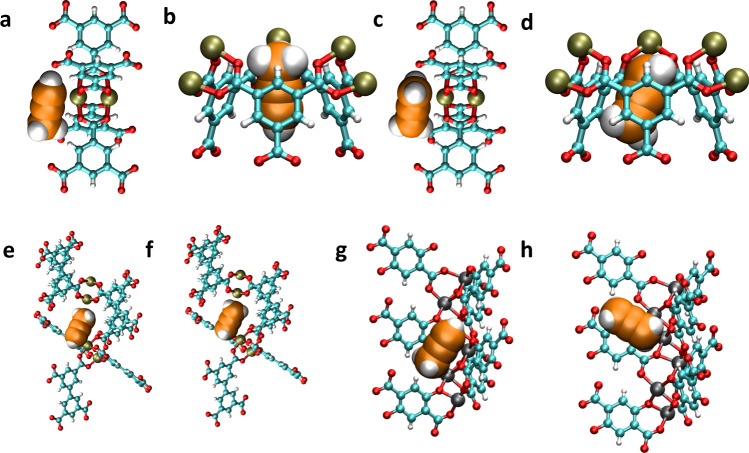
Perspective views of a portion of the crystal structure of HKUST-1, MOF-505, and Mg-MOF-74, showing the optimized position of propyne and propadiene molecules. **a** OMSs units of HKUST-1 for propyne. **b** Tetrahedral cages units of HKUST-1 for propyne. **c** OMSs units of HKUST-1 for propadiene. **d** Tetrahedral cages units of HKUST-1 for propadiene. **e** OMSs units of MOF-505 for propyne. **f** OMSs units of MOF-505 for propadiene. **g** OMSs units of Mg-MOF-74 for propyne. **h** OMSs units of Mg-MOF-74 for propadiene. Atom colors: C(MOF) = cyan, C(propene) = orange, H = white, N = blue, O = red, Cu = oliver green, Mg = sliver.

For the purpose of gaining insight into the nature of the binding sites and energetics for propyne and propadiene in microporous MOFs with OMSs, we conducted classical Monte Carlo (MC) simulations (details are provided in Supplementary Notes 5). These simulations revealed that propyne and propadiene adsorb at two main binding sites in HKUST-1: (a) the Cu OMSs and (2) between tetrahedral cages. These are denoted as sites I and II, respectively (Figs. 7a–d). Calculation of the averaged classical potential energy for propyne and propadiene adsorbed at the site I in HKUST-1 afforded values of −54.15 and −50.74 kJ mol^−1^, respectively (Supplementary Table 6). Notably, there are strong electrostatic and polarizable interactions between the positively charged Cu^2+^ ions of the copper paddlewheels and the unsaturated C atoms of the adsorbate molecules. The region between the tetrahedral cages is also favorable for propyne and propadiene, mainly due to strong π–π interactions between the aromatic rings of BTC linkers and the adsorbates. The averaged classical potential energy for propyne and propadiene adsorbed at site II in HKUST-1 were calculated to be −47.37 and −48.85 kJ mol^−1^, respectively (Supplementary Table 6). The potential energy for propadiene at site II is slightly higher than that for propyne, presumably because it can make more contacts with the framework within this region due to having two π bonds positioned over three atoms (compared to two π bonds centered on two atoms in propyne). In general, the calculated potential energies for propyne and propadiene about both sites in HKUST-1 are comparable in magnitude to the *Q*_st_ values for the respective adsorbates (Supplementary Fig. 16a). Figure 7e, f shows the most favorable binding site for propyne and propadiene, respectively, in MOF-505 as determined through classical MC simulations. As with HKUST-1, both adsorbates bind to Cu^2+^ ions of the [Cu_2_(O_2_CR)_4_] units. The averaged classical potential energies for propyne and propadiene about the OMSs were calculated to be −89.17 and −43.19 kJ mol^−1^, respectively (Supplementary Table 6). These values are comparable to the initial Qst values calculated for MOF-505 (Supplementary Fig. 16b). The potential energy for propyne is much greater than that for propadiene presumably because its longer length (6.51 vs. 6.13 Å, Fig. 1b) allows for simultaneous interaction between the Cu^2+^ ions of adjacent copper paddlewheels. In Mg-MOF-74, the exposed Mg^2+^ ions are preferential binding sites for propyne and propadiene (Fig. 7g, h). Similar to the Cu^2+^ binding sites in HKUST-1 and MOF-505, favorable interactions occur between positively charged Mg^2+^ ions and the negatively charged unsaturated C atoms of the adsorbate molecules. The averaged classical potential energy for propyne localized at this site is greater than that for propadiene (−78.46 and −60.62 kJ mol^−^^1^, Supplementary Table 6). The calculated potential energy difference between propyne and propadiene in Mg-MOF-74 is similar to the difference in the initial *Q*_st_ values for both adsorbates (Supplementary Fig. 16c). It is known from our previous reports that NKMOF-1-Ni has a stronger affinity for propyne than propadiene^46^. Moreover, compared with HKUST-1 and MOF-505, Mg-MOF-74 and NKMOF-1-Ni have similar simulated breakthrough separation effects (Fig. 6b). But the results of the dynamic breakthrough experiments (Fig. 4) were less than satisfactory as the retained time of Mg-MOF-74 and NKMOF-1-Ni is much less than that of HKUST-1 and MOF-505. These results suggest that co-adorption or lack thereof may play a key role in the separation process.

### Kinetic adsorption study

**Fig. 8:**
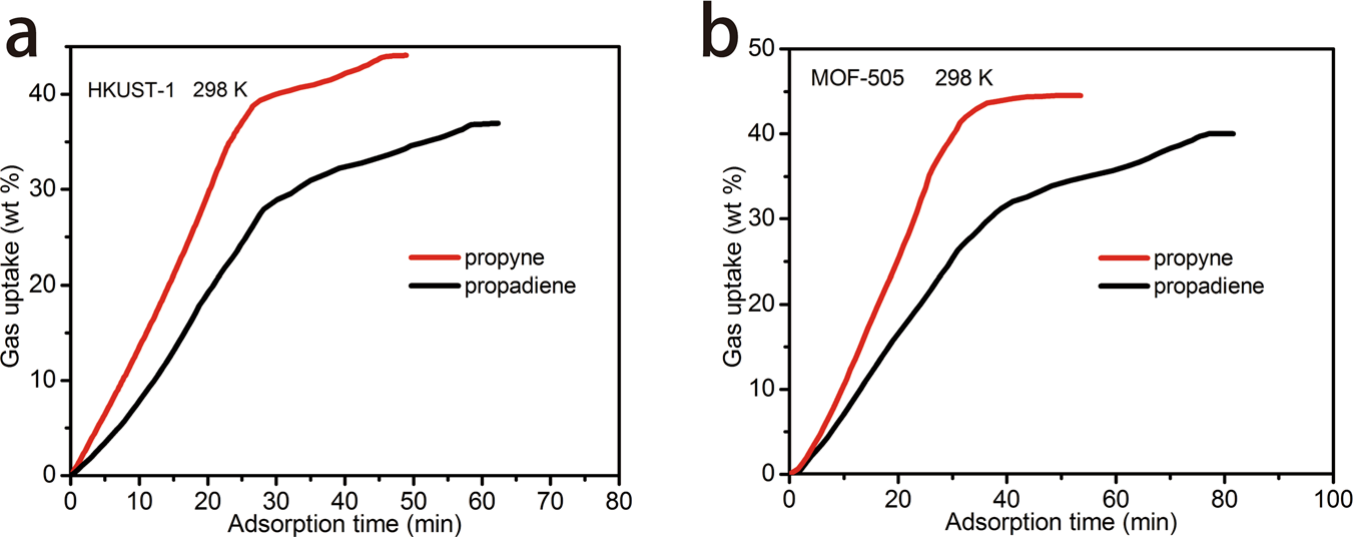
Gravimetric propyne and propadiene sorption kinetics at 298 K. **a** HKUST-1. **b** MOF-505.

Considering the difference between the actual and the simulated breakthrough experiments, we measured the kinetic adsorption behavior of HKUST-1, MOF-505, Mg-MOF-74, and NKMOF-1-Ni (Fig. 8 and Supplementary Fig. S17). The adsorption rates of propyne in HKUST-1 and MOF-505, which comprised OMSs and microporous cage-based molecule trap structures, were observed to be faster than that of propadiene (Fig. 8a, b). However, for Mg-MOF-74 and NKMOF-1-Ni, which comprised OMSs and smooth one-dimensional channels, the kinetic adsorption curves of propyne and propadiene almost overlapped and crossed without noticeable selection difference (Supplementary Fig. 17a, b). The dynamic adsorption differences may be linked to cage-based molecule traps. HKUST-1 and MOF-505 are comprised of two kinds of cages with different sizes and shapes (Supplementary Fig. 11). In this way, the diffusion rate of different gas molecules in the process of adsorption will be affected by the cage windows, thus explaining the diffusion rate of propyne vs. propadiene. For Mg-MOF-74 and NKMOF-1-Ni, which are comprised of open one-dimensional channels, gas molecules can freely diffuse in the channels. Moreover, whereas both propyne and propadiene strongly with OMSs in the channels of Mg-MOF-74 and NKMOF-1-Ni, their adsorption kinetics were similar. HKUST-1 and MOF-505 both exhibited benchmark separation performance for propyne/propadiene thanks to the synergy between kinetics and thermodynamics.

In summary, our study emphasizes the importance of both kinetics and thermodynamics with respect to the separation performance of propadiene vs. propyne. Dynamic gas mixture breakthrough experiments revealed that MOFs with OMSs and cage-based molecule traps exhibited such synergy to afford benchmark separation performance (propadiene production up to 69.6 cm^3^/g, purity > 99.5%) whereas traditional porous materials such as activated carbon and zeolites showed no separation effect. Our study offers a design principle for sorbent selection along with a screening protocol that might be broadly applied for sorbent evaluation.

## Methods

### Preparation of MOF materials and characterization

All samples were synthesized according to Supplementary Method 2. PXRD test was conducted using microcrystalline samples on a Rigaku Ultima IV diffractometer (40 kV, 40 mA, Cu Kα1, 2*λ* = 1.5418 Å). The measured parameter included a scan speed of 2 (°)/min, a step size of 0.02 (°). All MOFs samples were tested by ASAP 2020 PLUS Analyzer (Micromeritics) with Dewar (liquid N_2_) and a homemade intelligent temperature control system (0–80 °C). The SEM images were obtained on HITACHI SU3500.

### Breakthrough experiment

The breakthrough experiments were performed at a flow rate of 2 mL/min (298 K, 1.01 bar) for the propyne/propadiene (50/50, v/v) mixture. The MOF powder was packed into Φ 4 × 150 mm stainless steel column and activated by blowing pure helium (He). The column stacking density and column voidages of all samples tested were controlled in a similar condition. The breakthrough set-up consisted of two same fixed-bed stainless steel columns. One was used as a test sample and the other as a blank control to stabilize the gas flow. Both columns were housed in a temperature control system. The flow rates of all gases were regulated by mass flow controllers. The composition of the efflux gas of the test sample column was monitored by gas chromatography (Shimadzu, GC 2030, FID-Flame Ionization Detector, detection limit 100 ppb).

The propadiene productivity (*q*) was calculated by integration of the breakthrough curves *f*(*t*). The purity of propadiene is higher or equal to a threshold value during the integration interval from *t*1 to *t*2

$$q=\frac{{C}_{i}({{{{{\rm{propadiene}}}}}})}{{C}_{i}\left({{{{{\rm{propyne}}}}}}\right)+{C}_{i}({{{{{\rm{propadiene}}}}}})}\times \left({\int }_{t2}^{t1}f(t){dt}\right)$$

### Kinetic adsorption isotherm test

The kinetic adsorption behavior of HKUST-1, MOF-505, Mg-MOF-74, and NKMOF-1-Ni was performed on thermogravimetric analysis Instruments (Q50). During the test, we controlled the gas flow rate of propyne and propadiene to be 20 cm^3^/min. The data was collected in the High-Resolution Dynamic mode with a sensitivity of 1.0, a resolution of 4.0, and the weight changes during propyne and propadiene gas adsorption step were monitored under the isothermal condition at 25 °C (298 K).

### *Q*_st_, IAST and transient breakthrough simulation calculation

The *Q*_st_ was determined from the unary isotherm by use of the Clausius–Clapeyron equation. IAST calculations were carried out for the following mixture 50/50 propyne/propadiene mixture at 298 K. Transient breakthrough simulations were carried out for 50/50 propyne/propadiene feed mixture at 298 K and 100 kPa.

## Supplementary information

Supplementary Information

## Data Availability

The authors declare that the data supporting the findings of this study are available within the article and its Supplementary Information.
