# Supplementary material for: Efficient propyne/propadiene separation by microporous crystalline physiadsorbents
Source: Nat Commun. 2021 Oct 1;12:5768. doi: 10.1038/s41467-021-25980-y (PMC8486746; doi:10.1038/s41467-021-25980-y)
Supplement: Supplementary file 1 — Supplementary Information [file 41467_2021_25980_MOESM1_ESM.pdf]

Supplementary Information for

Efficient Propyne/Propadiene Separation by Microporous Crystalline Physiadsorbents

Yun-Lei Peng, Ting Wang, Chaonan Jin, Cheng-Hua Deng, Yanming Zhao,Wansheng Liu, Katherine A. Forrest, Rajamani Krishna, Yao Chen, Tony Pham, Brian Space, Peng Cheng, Michael J. Zaworotko, Zhenjie Zhang*
*Correspondence to: zhangzhenjie@nankai.edu.cn (Z. Z.)

Supplementary Methods:

1. **General Materials**

1,3,5-Tricarboxybenzene (98%, innochem), 2,5-Dihydroxy-1,4-benzenedicarbo- -xylic acid (H_4DOBDC) (98%, innochem), Biphenyl-3,3',5,5'-tetracarboxylic Acid (99%, innochem), Pyrazine (99%, innochem), 1,4-Benzenedicarboxylic acid (99%, Macklin), Biphenyl-4,4’-dicarboxylic acid (97%, Heowns,Tianjin), 4,4’-azopyridine (95+%, HUAWEIRUIKE), 2,3-dichloropyrazine (98%, Bide Pharmatech Ltd), 4-(2-pyridin-4-ylethynyl), Pyridine (98%, HUAWEIRUIKE), 1-Methylimidazole (99%, aladdin), $Cu(NO_3)_2·3H_2O$ (Analytical reagent, Gerhardite), $ZrCl_4$ (99.9%, Aladdin), $Zn(NO_3)·6H_2O$ (99.9%, Heowns,Tianjin), $(NH_4)_2·SiF_6$ (Analytical reagent, Greagent), $CuSiF_6$ (Analytical reagent, Greagent), $Ni(BF_4)_2·6H_2O$ (Energy chemical), Sodium hydroxide (NaOH, 97%, Aladdin), Sodium hydrosulfide (NaHS, LiDeShi), Copper(II) perchlorate hexahydrate ($Cu(ClO_4)_2·6H_2O$, 98%, Strem Chemical, Inc.), Niobium Oxide (Nb_2O_5, 99.9%, Macklin), Nickel tetrafluoroborate hexahydrate ($Ni(BF_4)_2·6H_2O$, 99%, Aldrich), Chromic nitrate ($Cr(NO_3)_3$, 99%, Macklin), Chromium sesquioxide (Cr_2O_3, 99%, Macklin), Iron powder (99.95%, Aladdin), N,N-dimethyl-Formamide

(DMF), Ethanol,, Methanol, Acetonitrile, Ether, and Acetone were purchased and used without further purification. He, N_2, propyne ($CH_3C{\equiv}CH$), and propadiene ($CH_2{=}C{=}CH_2$), CO_2, were purchased from AIR LIQUIDE. The powder X-ray diffraction data were obtained on Rikagu ULTIMA IV.

2. **MOFs Synthesis**

Synthesis of HKUST-1: HKUST-1 was synthesized based on the previous method.[1] $Cu(NO_3){\cdot}2.5H_2O$ (6.0 g) was dissolved into 250 mL deionized water. It was followed by the addition of 1,3,5-Tricarboxybenzene (4.0 g) in a 250 mL of solvent consisting of equal parts of ethanol and deionized water and mixed thoroughly until it was completely dissolved. The resultant solution mixture was transferred into a 250 mL teflon-lined stainless steel autoclave. It was kept at 110 ℃ for 18 h in oven to yield small crystals. Then the autoclave was cooled down to room temperature naturally and the blue crystals were isolated by filtration. The suspension was washed with the mixture of deionized water and ethanol several times and dried.

Synthesis of MOF-505: MOF-505 was synthesized based on the previous method.[2] A solid mixture of Biphenyl-3,3',5,5'-tetracarboxylic Acid (H_4bptc; 25 mg, 0.076 mmol) and $Cu(NO_3)_2{\cdot}(H_2O)_{2.5}$ (52 mg, 0.22 mmol) was added 5ml mixture solvent (DMF/ethanol/H_2O 3/3/2 (v/v/v)) in 20 mL scintillation vials. The reaction vials were capped tightly with Teflon-lined caps and placed in an oven at 65℃ for 24 hours. Then the gave green, block shaped Crystals was yield.

Synthesis of Mg-MOF-74: Mg-MOF-74 was synthesized based on the previous method.[3] A solid mixture of H_4DOBDC (0.111 g, 0.559 mmol) and $Mg(NO_3)_2{\cdot}6H_2O$

(0.475 g, 1.85 mmol) was added 50 ml mixture solvent (DMF/ethanol/water 15/1/1 (v/v/v)). The suspension was mixed and ultrasonicated until homogeneous. The reaction solution was then dispensed to five 20 mL scintillation vials. The reaction vials were capped tightly with Teflon-lined caps and placed in an oven at 125 °C for 20 hours. Then the samples were removed from the oven and allowed to cool to RT. The mother liquor was decanted from the yellow microcrystalline material and replaced with methanol (10 mL per vial). The yellow microcrystalline material was combined into one vial. The methanol was decanted and replenished four times over two days. The solvent was removed under vacuum at 250 °C over 10 hours, yielding the dark yellow microcrystalline, porous material.

Synthesis of NKMOF-1-Ni: NKMOF-1-Ni was synthesized based on the previous method.[4] CuI (0.2143 g, 1.125 mmol) was dissolved in acetonitrile (30 mL) by sonication. $Na[Ni(pdt)_2]\cdot 2H_2O$ (0.30 g, 0.75 mol) was dissolved in acetonitrile (200 mL) and bubbled with argon for 1 hour. Subsequently, CuI solution was dropwise added into $Na[Ni(pdt)_2]\cdot 2H_2O$ solution. After 2 hours, dark red powders of NKMOF-i-Ni were obtained by centrifugation.

Synthesis of MIL-100 (Cr, Fe): MIL-100 (Cr) was synthesized based on the previous method.[5] Metallic chromium (52 mg, 1 mmol) was dispersed into an aqueous solution of 5M hydrofluoric acid (0.4 mL, 2 mmol). After the addition of 1,3,5-benzene tricarboxylic acid (H_3BTC) (150 mg, 0.67 mmol) and H_2O (4.8 mL, 265 * 10^{-3}mol), the mixture was heated in ahydrothermal bomb at a rate of 20 ℃/h to 220 ℃, kept at this temperature during 96 h, then cooled at a rate of 10 ℃/h to room temperature. The

resulting green powder was washed with deionized water and acetone and dried in air.

MIL-100 (Fe) was synthesized based on the previous method.[6] This solid was isolated as a polycrystalline powder from a reaction mixture of composition Fe/ H_3BTC /HF/HNO_3/H_2O (1.0/0.66/2.0/1.2/280) that was held at 150 ℃ in a Teflon-lined autoclave for 6 days with a initial heating ramp of 12 h and a final cooling ramp of 24 h. The pH remains acidic (<1) throughout the synthesis. The light-orange solid product was recovered by filtration and washed with deionized water. A treatment in hot deionised water (80 ℃) for 3 h was applied to decrease the amount of residual H_3BTC (typically, 1 g of MIL-100(Fe) in 350 ml of water) followed by drying at room temperature.

Synthesis of MIL-101 (Cr): MIL-101 (Cr) was synthesized based on the previous method.[7] A typical synthesis involves a solution containing chromium(III) nitrate $Cr(NO_3)_3 \cdot 9H_2O$ (400 mg, 1 mmol, 1 mmol of fluorhydric acid, 1,4-benzene dicarboxylic acid H_2BDC (164 mg, 1 mmol) in 4.8 ml H_2O (265 mmol); the mixture is introduced in a hydrothermal bomb which is put during 8h in an autoclave held at 220°C. A treatment in hot deionised water (80 °C) for 3 h was applied to decrease the amount of residual H_3BTC followed by drying at room temperature.

Synthesis of SIFSIX-2-Cu-i: SIFSIX-2-Cu-i was synthesized based on the previous method.[8] A methanol solution of 1,2-bis(4-pyridyl)acetylene (dpa) (4 mL, 0.270 mmol) was stirred with an aqueous solution of $CuSiF_6$ (4 mL, 0.258 mmol) resulting in a purple precipitate, which was then heated at 85°C for 12 hrs.

Synthesis of SIFSIX-3-Ni: SIFSIX-3-Ni was synthesized based on the previous

method.[9] SIFISIX-3-Ni was synthesized by slurrying 870 mg (3 mmol) of $Ni(NO_3)_2$, 534 mg (3 mmol) of $(NH_4)_2SiF_6$ and 480 mg (6 mmol) of pyrazine in 4 mL of water for 2 days. The resulting suspension was filtered under vacuum and dried in air. This precursor was soaked in methanol for 1 day and then washed twice with two portions (ca. 10 mL) of methanol on a Buchner filter. After drying in air, the solid was heated at 140 ˚C for 1 day to obtain SIFSIX-3-Ni.

Synthesis of UTSA-200 (SIFSIX-14-Cu-i): UTSA-200 (SIFSIX-14-Cu-i) was synthesized based on the previous method.[10] A methanol solution (3 mL) of azpy (0.266 mmol) was mixed with an aqueous solution of $CuSiF_6$ (2.5 mL, 0.247 mmol) at 80°C resulting in a bright grey precipitate, which was then heated at 80°C for 15 min, additional 1 h at 50°C, and then at room temperature for 24 h.

Synthesis of ZU-62: ZU-62 was synthesized based on the previous method.[11] A preheated water solution (4.0 mL) of $CuNbOF_5$ (0.0730 g) were dropped into a preheated methanol solution (4.0mL) of 4, 4'-bipyridylacetylene (0.0515 g). Then the mixture was heated at 80 °C for 24 h. The obtained blue power was exchanged with methanol for a day.

Synthesis of UiO-66: UiO-66 was synthesized based on the previous method.[12] Standard synthesis of UiO-66 was performed by dissolving $ZrCl_4$ (0.053 g, 0.227 mmol) and 1,4-benzenedicarboxylic acid (H_2BDC) (0.034g, 0.227 mmol) in DMF (24.9 g, 340 mmol) at room temperature. The thus obtained mixture was sealed and placed in a pre-heated oven at 120 ˚C for 24 hours. Crystallization was carried out under static conditions. After cooling in air to room temperature the resulting solid was filtered,

repeatedly washed with DMF and dried at room temperature.

Synthesis of UiO-67: UiO-67 was synthesized based on the previous method.[12] Standard synthesis of UiO-67 was performed by dissolving $ZrCl_4$ (0.053 g, 0.227 mmol) and 1,4-benzenedicarboxylic acid (H_2BPDC) (0.227 mmol) in DMF (24.9 g, 340 mmol) at room temperature. The thus obtained mixture was sealed and placed in a pre-heated oven at 120 ˚C for 24 hours. Crystallization was carried out under static conditions. After cooling in air to room temperature the resulting solid was filtered, repeatedly washed with DMF and dried at room temperature.

Synthesis of ZIF-8: ZIF-8 was synthesized based on the previous method.[13] A solid mixture of zinc nitrate tetrahydrate $Zn(NO_3)_2\cdot 4H_2O$ (0.210 g, 0.803 mmol) and 2-methylimidazole (H-MeIM) (0.060 g, 0.731 mmol) was dissolved in 18 mL DMF in a 20-mL vial. The vial was capped and heated at a rate of 5 ºC /min to 140 ºC in a programmable oven, held at this temperature for 24 h, then cooled at a rate of 0.4 ºC/min to room temperature. After removal of mother liquor from the mixture, chloroform (20 mL) was added to the vial. Colorless polyhedral crystals of the product were collected from the upper layer, washed with DMF (10 mL × 3) and dried in air (10 min).

Supplementary Notes:

Supplementary Note 1: Fitting of experimental data on pure component isotherm

The unary isotherm data for propyne and propadiene in HKUST-1 and Mg-MOF-74 (at 273 K and 298 K) were individually fitted with the dual-site Langmuir (DSL) model:

$$q = \frac{q_{sat,A} b_A p}{1 + b_A p} + \frac{q_{sat,B} b_B p}{1 + b_B p}$$ **Supplementary Equation 1**

The isotherm fit parameters for HKUST-1 are provided in Supplementary Table 2. The isotherm fit parameters for Mg-MOF-74 are provided in Supplementary Table 3.

The unary isotherm data for propyne and propadiene in MOF-505 (at 273 K and 298 K) and NKMOF-1-Ni (at 298 K and 318 K) were individually fitted with the dual-site Langmuir-Freundlich (DSLF) model:

$$q = \frac{q_{sat,A} b_A p^{V_a}}{1 + b_A p^{v_A}} + \frac{q_{sat,B} b_B p^{v_B}}{1 + b_B p^{v_B}}$$ **Supplementary Equation 2**

The isotherm fit parameters for MOF-505 are provided in Supplementary Table 4. The isotherm fit parameters for NKMOF-1-Ni are provided in Supplementary Table 5.

Supplementary Note 2: Isosteric heat of adsorption

The isosteric heat of adsorption was determined from the unary isotherm by use of the Clausius-Clapeyron equation:

$$Q_{st} = RT^2 \left(\frac{\partial 1np}{\partial T} \right)_q$$ **Supplementary Equation 3**

These values were determined using the pure component isotherm fits using the DSL and DSLF equation (Supplementary Fig. 12-14). $\boldsymbol{Q_{st}}$ is the coverage dependent isosteric heat of adsorption and ***R*** is the universal gas constant.

Supplementary Note 3: IAST calculations of adsorption selectivity and uptake capacity

IAST calculations were carried out for the following mixture 50/50 propyne/propadiene mixture at 298 K. the adsorption selectivity is defined by:

$$S_{ads} = \frac{q_A / q_B}{y_A / y_B}$$ **Supplementary Equation 4**

Where the $\boldsymbol{q_A}$, and $\boldsymbol{q_B}$ represent the molar loadings within the MOF that is in equilibrium with a bulk fluid mixture with mole fractions $\boldsymbol{y_A}$, and $\boldsymbol{y_B}$ =1 - $\boldsymbol{y_A}$. The molar loadings, also called gravimetric uptake capacities, are usually expressed with the units mol Kg^{-1}. The IAST calculations of propyne/propadiene adsorption selectivites taking the mole fractions $\boldsymbol{y_A}$ = 0.5 and $\boldsymbol{y_B}$ = 1 - $\boldsymbol{y_A}$ = 0.5 for a total pressure of 100 kPa and 298 K.

Supplementary Note 4: Transient breakthrough simulation

The performance of industrial fixed bed adsorbers is dictated by a combination of adsorption selectivity and uptake capacity. Transient breakthrough simulations were carried out for 50/50 propyne/propadiene feed mixture at 298 K and 100 kPa using the methodology described in earlier publications.[14-18] For the breakthrough simulations, the following parameter values were used: length of packed bed, L = 0.3 m; voidage of packed bed, ε = 0.4; superficial gas velocity at inlet, u = 0.04 m/s. The y-axis is the dimensionless concentrations of each component at the exit of the fixed bed, c_i / c_{i0}

normalized with respect to the inlet feed concentrations. The x-axis is the dimensionless time, $\tau = \frac{tu}{L\varepsilon}$, defined by dividing the actual time, *t*, by the characteristic time, $\frac{L\varepsilon}{u}$.

Notation

b	Langmuir-Freundlich parameter,
q	component molar loading of species i, mol kg^{-1}
q_{sat}	saturation loading, mol kg^{-1}
L	length of packed bed adsorber, m
t	time, s
T	absolute temperature, K
u	superficial gas velocity in packed bed, m s^{-1}

Greek letter

ε	voidage of packed bed, dimensionless
ν	Freundlich exponent, dimensionless
τ	time, dimensionless

Supplementary Note 5: Modeling Study

All parametrizations and simulations in HKUST-1 and MOF-505 were performed on the single X-ray crystallographic structure of the materials as published in references (CCDC 112954)[19] and (CCDC 257470),[20]]respectively. The polarizable force field that was developed for Mg-MOF-74 in previous work was used herein.[21]

All atoms of HKUST-1 and MOF-505 were treated with Lennard-Jones (LJ) parameters (ε and σ), point partial charges,[22] and point polarizabilities in order to model repulsion/dispersion, stationary electrostatic, and many-body polarization interactions, respectively. The LJ parameters for all atoms were taken from the Universal Force Field (UFF).[23] The partial charges for the chemically distinct atoms in both MOFs were determined through electronic structure calculations on different gas phase fragments that were selected from the crystal structure of the respective MOFs. These calculations were performed using the NWChem *ab initio* software with the 6-31G* basis set assigned to C, H, and O and the LANL2DZ ECP basis set assigned to Cu.[24-27] The exponential damping-type polarizability values for all C, H, and O atoms were taken from a carefully parametrized set provided by the work of van Duijnen and Swart.[28] The polarizability parameter for Cu^{2+} was calculated in previous work and used herein.[29]

Classical Monte Carlo (MC) simulations of propyne and propadiene adsorption were performed within the unit cell of HKUST-1, 2 × 2 × 1 supercell of MOF-505, and 1 × 1 × 4 supercell of Mg-MOF-74. All MOF atoms were kept fixed at their crystallographic positions. For each MOF, a spherical cut-off distance corresponding to half the shortest system cell dimension length was used for the simulations. Propyne and propadiene were modeled using polarizable potentials of the respective adsorbates that were developed previously.[30] The total potential energy of the MOF-adsorbate system was calculated through the sum of the repulsion / dispersion, stationary electrostatic, and many-body polarization energies. These were calculated using the

Lennard-Jones 12-6 potential,[22] partial charges with Ewald summation, [31,32] and a Thole-Applequist type model,[33-36] respectively. All MC simulations were performed using the Massively Parallel Monte Carlo (MPMC) code. [37,38]

In order to identify the global minimum for propyne and propadiene in HKUST-1, MOF-505, and Mg-MOF-74, simulated annealing (SA) calculations were performed for a single molecule of each adsorbate through a Canonical Monte Carlo (CMC) process in the considered system cell of the individual MOFs.[39] All SA calculations utilized an initial temperature of 500 K, and this temperature was scaled by a factor of 0.99999 after every 1.0×10^3 MC steps. The simulations continued until 1.0×10^6 MC steps were reached; at this point, the temperature of the system is below 10 K and the adsorbate is already localized in its energy minimum position in the MOF. For all three MOFs, the global minimum for propyne and propadiene was observed to be localization onto the open-metal sites. Additional SA calculations in HKUST-1 revealed that propyne and propadiene also settled into the tetrahedral cages, which represent the local minimum (i.e., secondary binding site) in the material.

Next, CMC simulations were performed for a single molecule of propyne and propadiene, individually, positioned at their global minimum in all three MOFs.[40] This was done in order to evaluate the averaged classical potential energy for both adsorbates about their energy minimum position in the respective materials. The CMC simulations were performed at a temperature of 298 K and a pressure of 0.10 atm. These simulations ran for a total of 1.0×10^6 MC steps to ensure reasonable ensemble averages for the total potential energy of the system. Similar CMC simulations were carried out for both

adsorbates initially positioned within the tetrahedral cage in HKUST-1. The averaged classical potential energies for propyne and propadiene localized about the favorable binding sites in HKUST-1, MOF-505, and Mg-MOF-74 are presented in Supplementary Table 6.

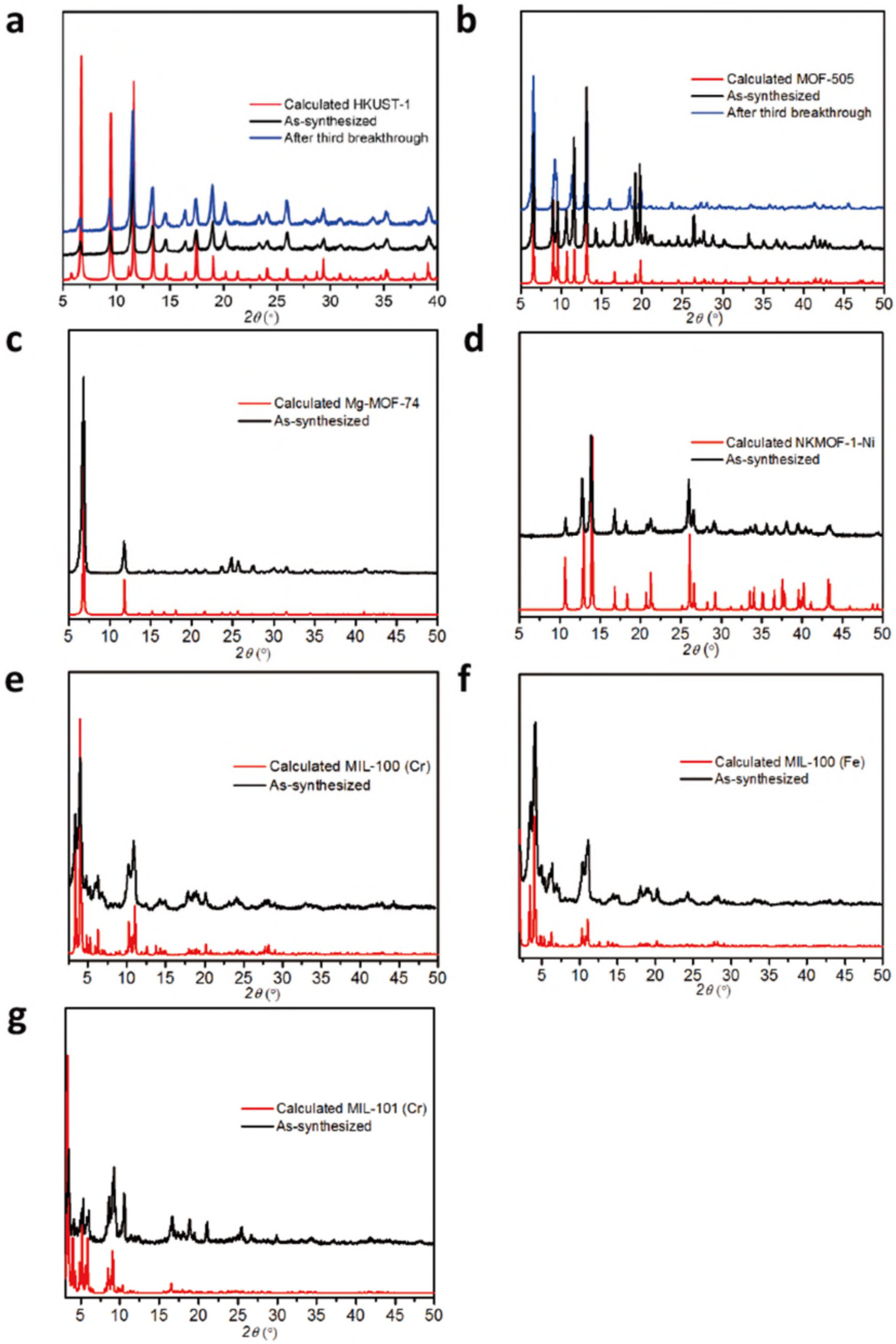

Supplementary Fig. 1 The PXRD patterns of the MOFs with open metal sites (OMSs). **a** HKUST-1. **b** MOF-505. **c** Mg-MOF-74. **d** NKMOF-1-Ni. **e** MIL-100(Cr). **f** MIL-100(Fe). **g** MIL-101.

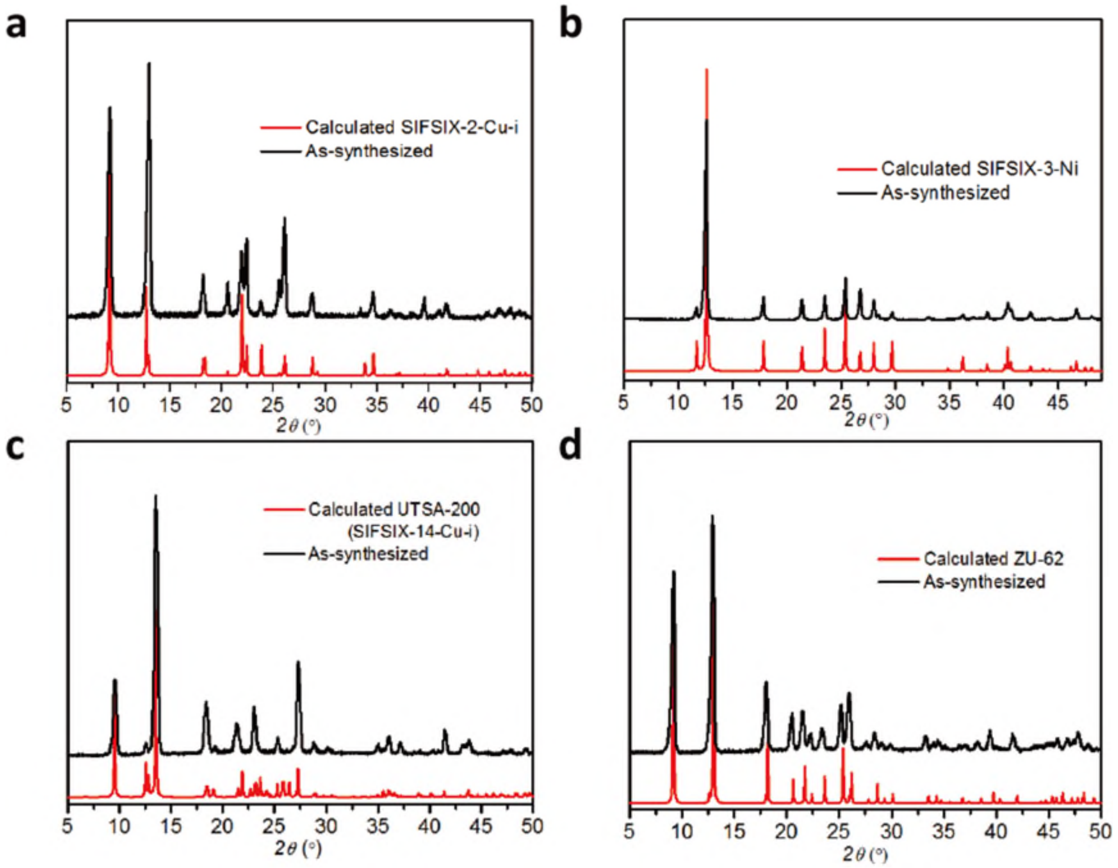

Supplementary Fig. 2 The PXRD patterns of the MOFs with strong binding sites. **a** SIFSIX-2-Cu-i. **b** SIFSIX-3-Ni. **c** UTSA-200. **d** ZU-62.

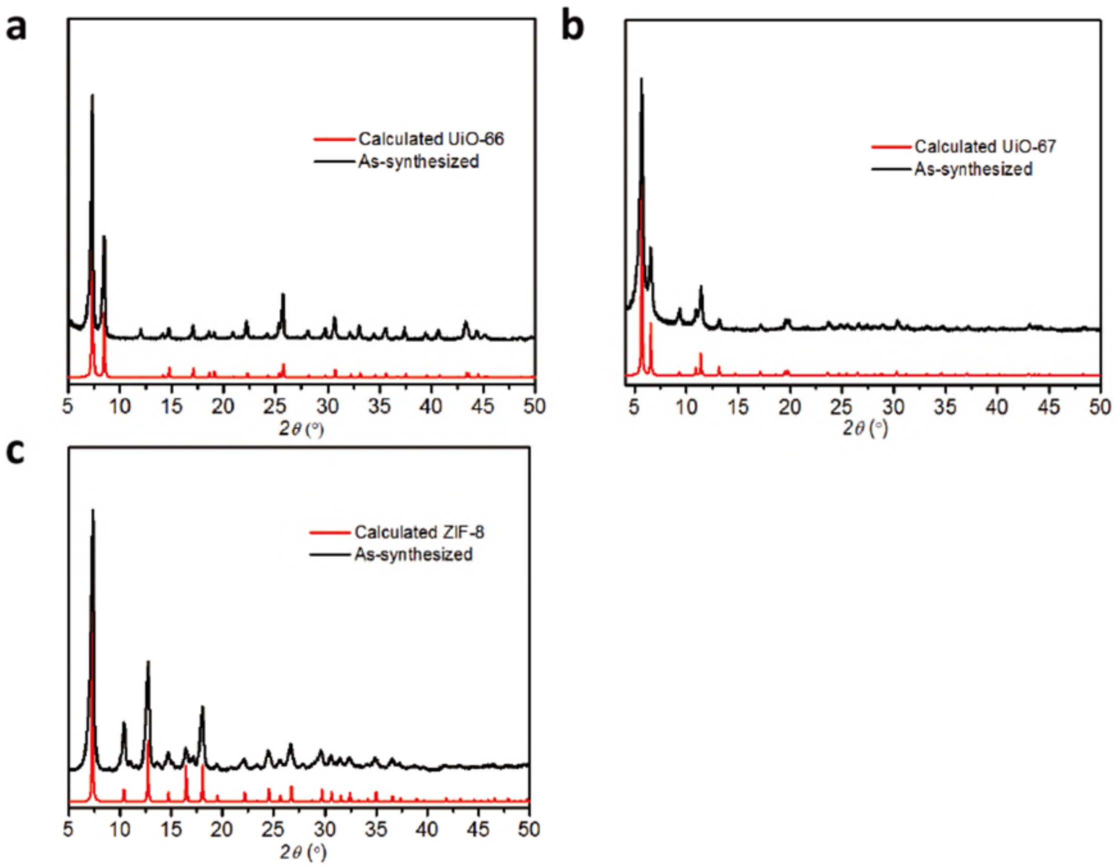

Supplementary Fig. 3 The PXRD patterns of the MOFs without strong binding sites. **a** UiO-66. **b** UiO-67. **c** ZIF-8.

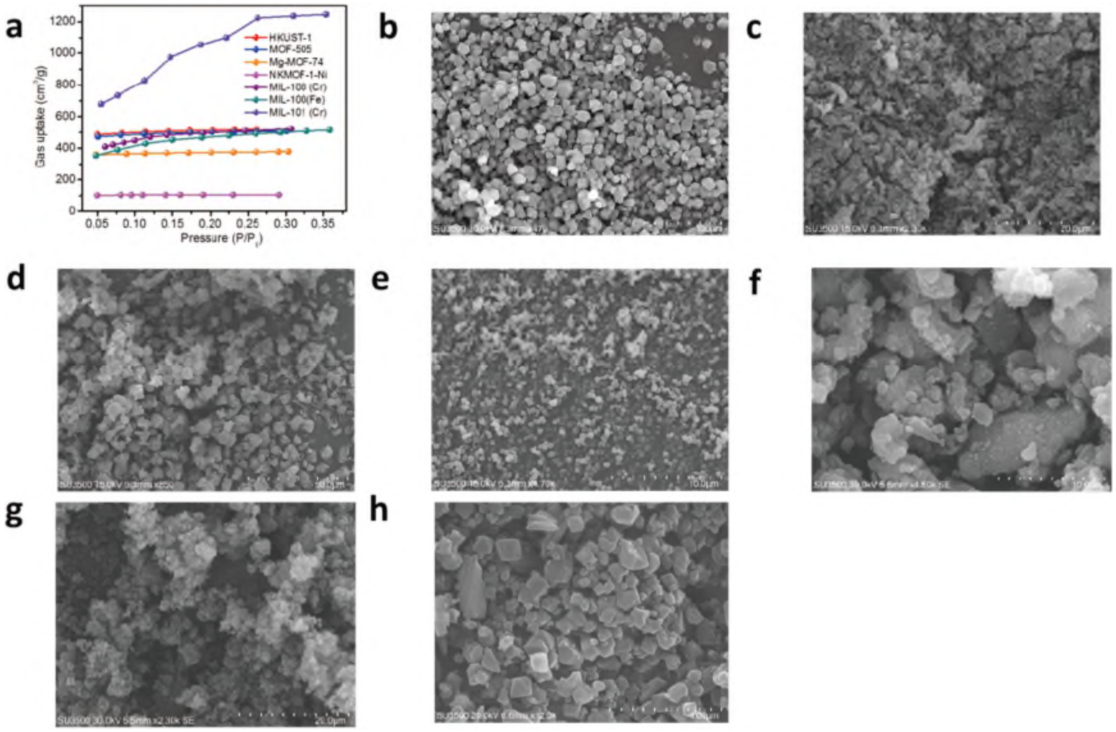

Supplementary Fig. 4 BET and SEM measurements for MOFs with open metal sites. **a** N_2 adsorption of MOFs with open metal sites at 77K. **b-h** The SEM images for HKUST-1, MOF-505, Mg-MOF-74, NKMOF-1-Ni, MIL-100(Cr), MIL-100(Fe) and MIL-101(Cr).

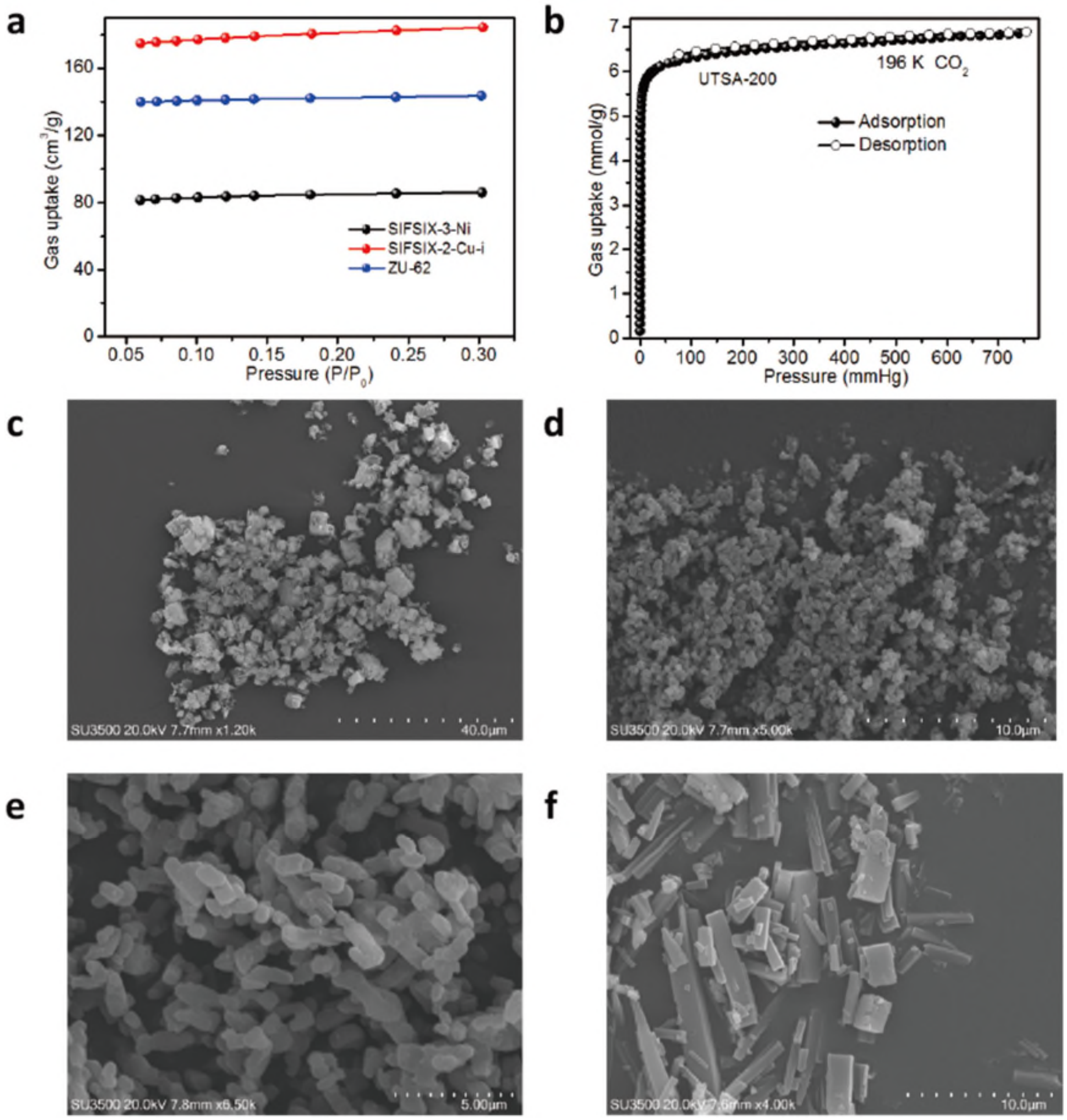

Supplementary Fig. 5 BET and SEM measurements for MOFs with strong binding sites. **a** N_2 adsorption of SIFSIX-3-Ni, SIFSIX-2-Cu-i and ZU-62 at 77K. **b** CO_2 adsorption of UTSA-200 at 196K. **c-f** The SEM images for SIFSIX-3-Ni, SIFSIX-2-Cu-i, ZU-62 and UTSA-200.

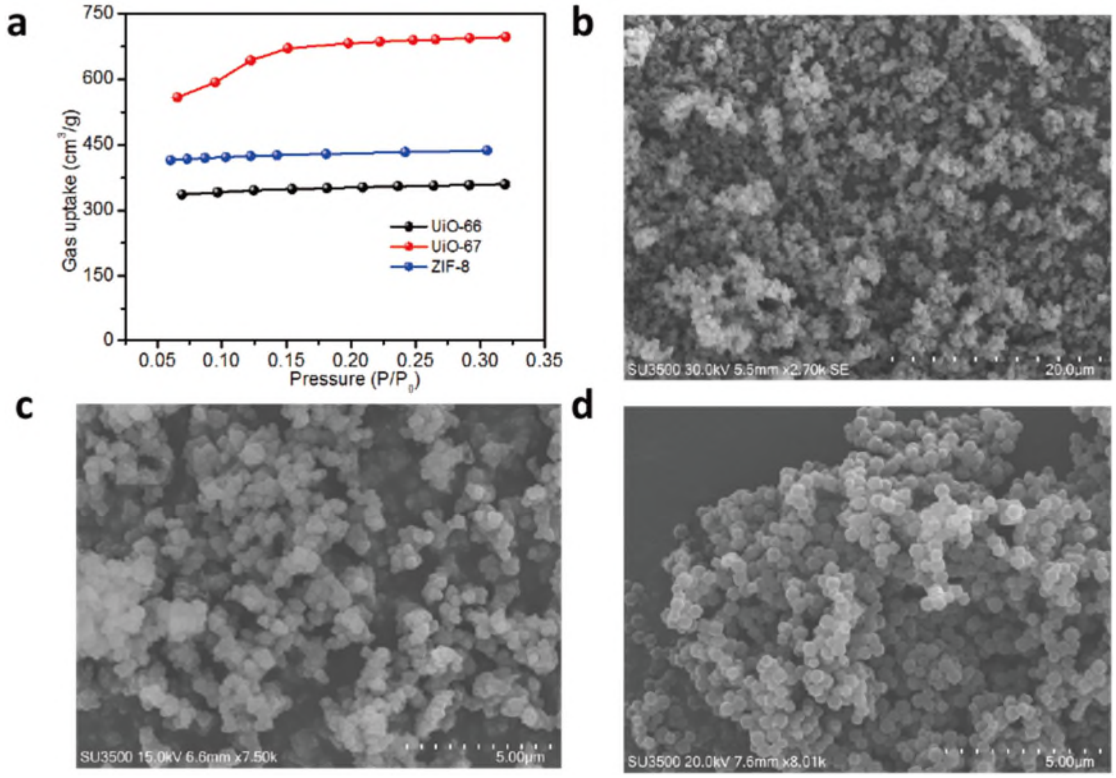

Supplementary Fig. 6 BET measurements for MOFs without strong binding sites. **a** N_2 adsorption of UiO-66, UiO-67 and ZIF-8 at 77K. **b-d** The SEM images for UiO-66, UiO-67 and ZIF-8.

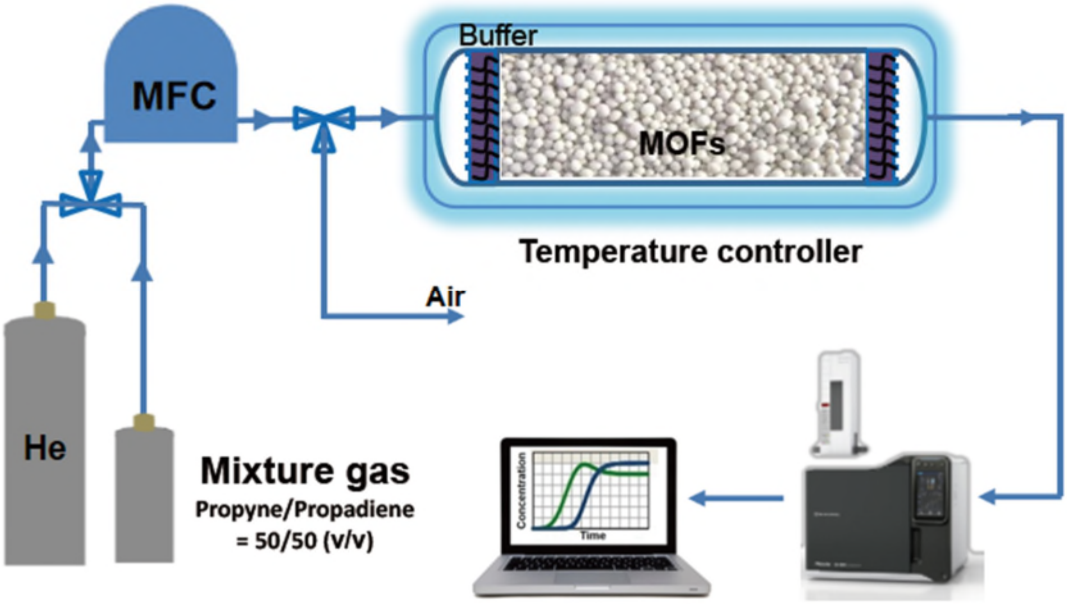

Supplementary Fig. 7 Breakthrough separation apparatus. (MFC= Mass Flow Controller)

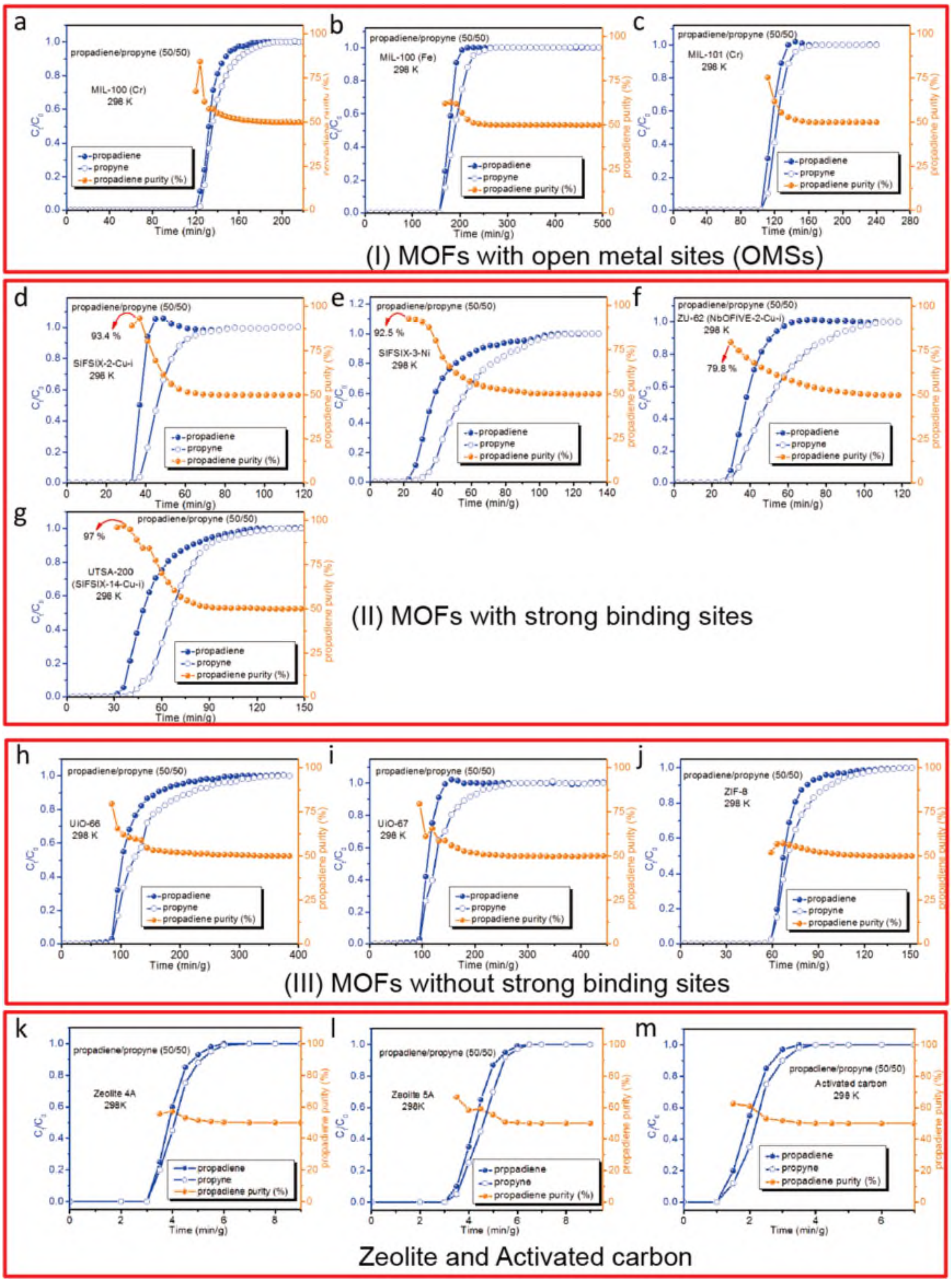

Supplementary Fig. 8 Experimental breakthrough curves of the selected MOF materials for propyne/propadiene (50/50, v/v) at 298 K. **a**, **b**, **c** MOFs with open metal sites (MIL-100(Cr), MIL-100 (Fe) and MIL-101 (Cr)). **d**, **e**, **f**, **g** MOFs with strong binding sites (SiF_6^{2-} and $NbOF_5^{2-}$) (SIFSIX-2-Cu-i, SIFSIX-3-Ni, ZU-62, and UTSA-200). **h**, **i**, **j** some MOFs without strong binding sites (UiO-66, UiO-67, and ZIF-8). **k** Zeolite 4A. **l** Zeolite 5A. **m** Activated carbon.

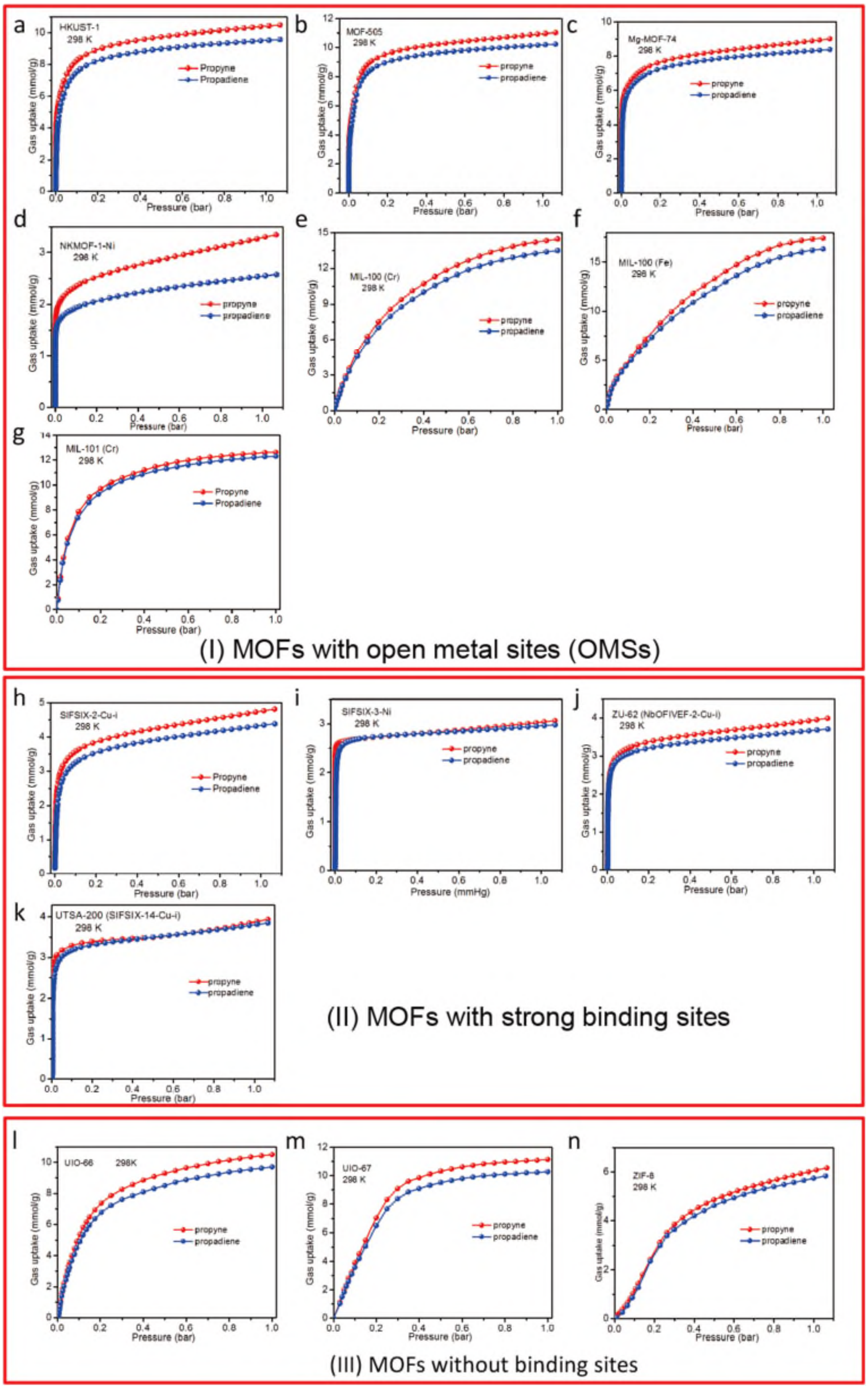

Supplementary Fig. 9 Experimental propyne (red) and propadiene (blue) adsorption isotherms of the selected MOF materials at 298 K. **a**, **b**, **c**, **d**, **e**, **f**, **g** MOFs with open metal sites (HKUST-1, MOF-505, Mg-MOF-74, NKMOF-1-Ni, MIL-100(Cr), MIL-100(Fe) and MIL-101(Cr)). **h**, **i**, **j, k** MOFs with strong binding sites (SiF_6^{2-} and $NbOF_5^{2-}$ sites) (SIFSIX-2-Cu-i, SIFSIX-3-Ni, ZU-62, and UTSA-200). **l**, **m, n** MOFs without binding sites (UiO-66, UiO-67 and ZIF-8).

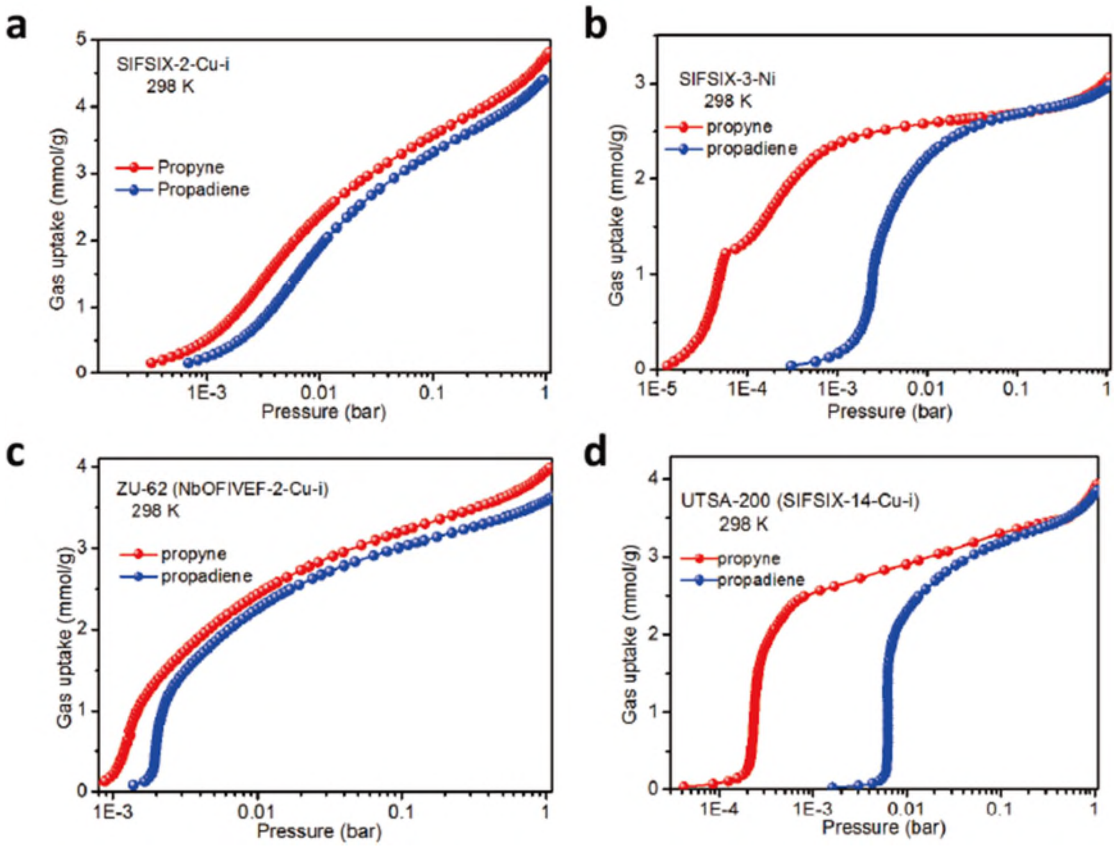

Supplementary Fig. 10 propyne (red) and propadiene (blue) equilibrium adsorption isotherms of MOFs with strong binding sites. **a** SIFSIX-2-Cu-i. **b** SIFSIX-3-Ni. **c** ZU-62. **d** UTSA-200.

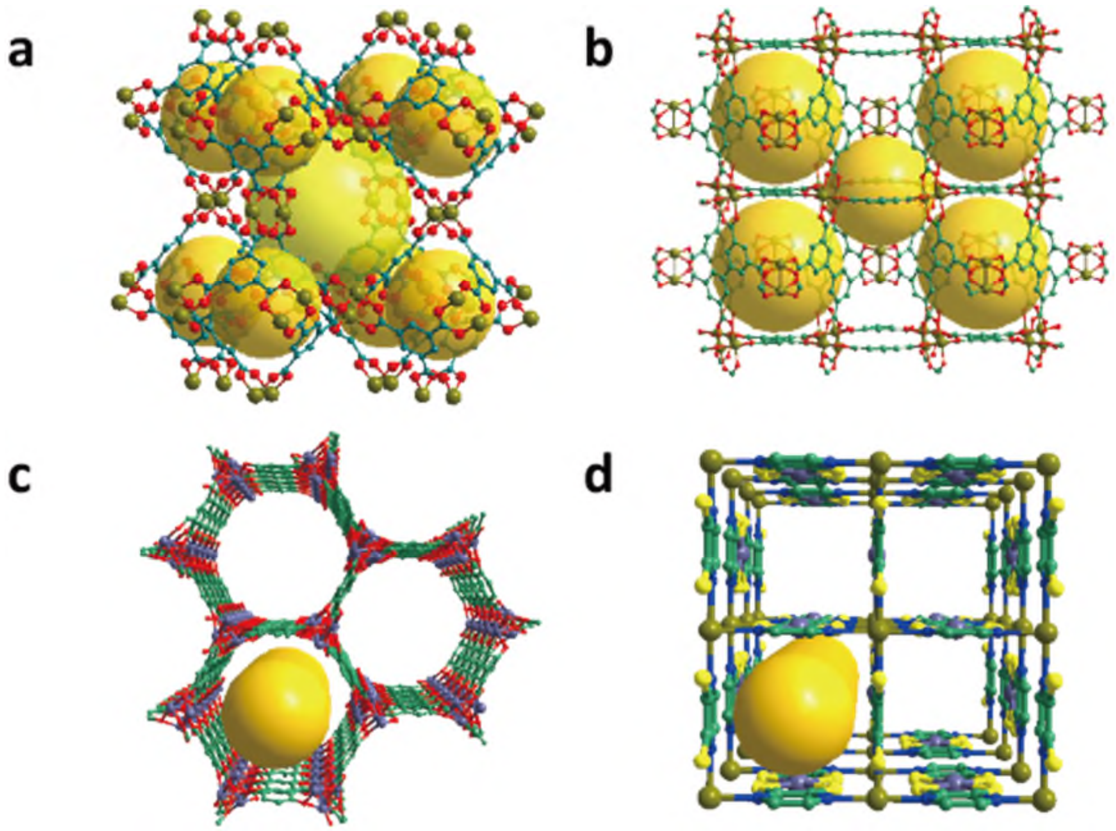

Supplementary Fig. 11 The schematic of microporous MOFs with OMSs. **a, b** HKUST-1 and MOF-505 with OMSs and cage-based structure. **c, d** Mg-MOF-74 and NKMOF-1-Ni with OMSs and smooth one-dimensional channels. Atom colors: C(MOF) = sea green, N = blue, O = red, Cu = dark yellow, Ni = sliver, Mg = blue gray.

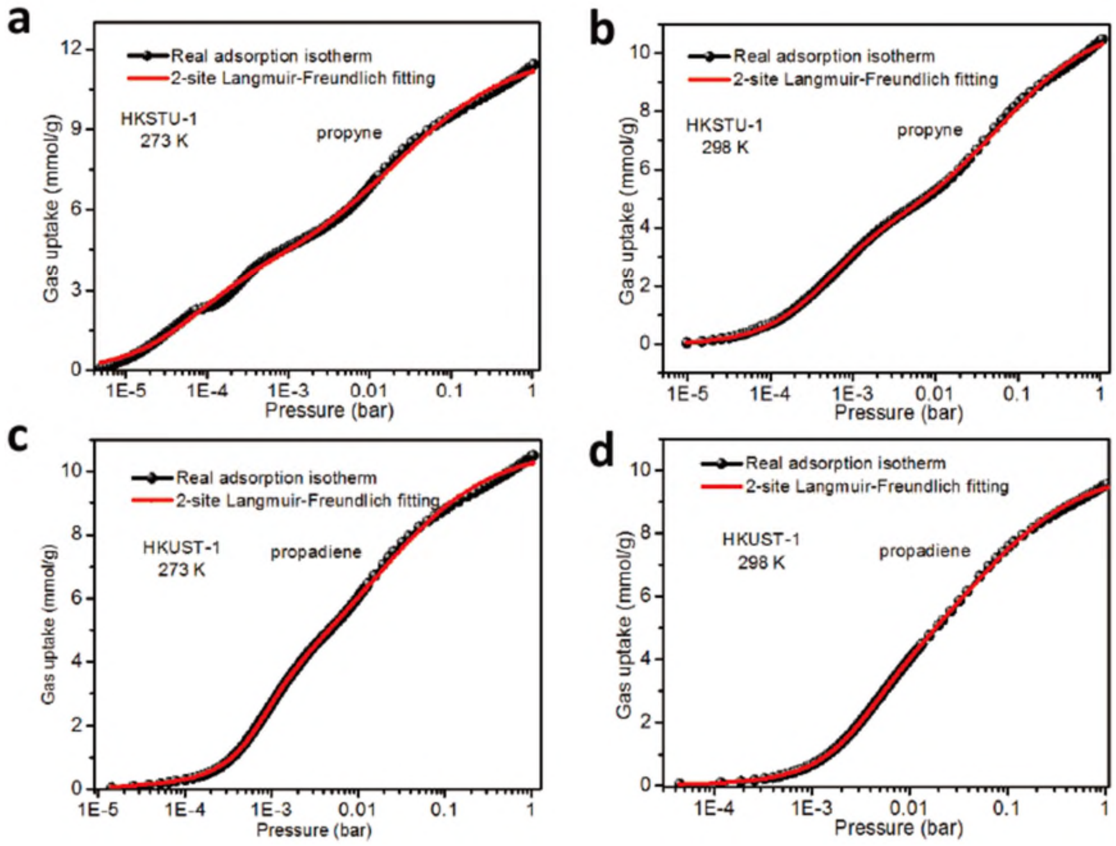

Supplementary Fig. 12 Dual-site Langmuir-Freundlich model fits of propyne and propadiene adsorption isotherms at 273 K and 298 K in HKUST-1. **a, b** propyne. **c, d** propadiene.

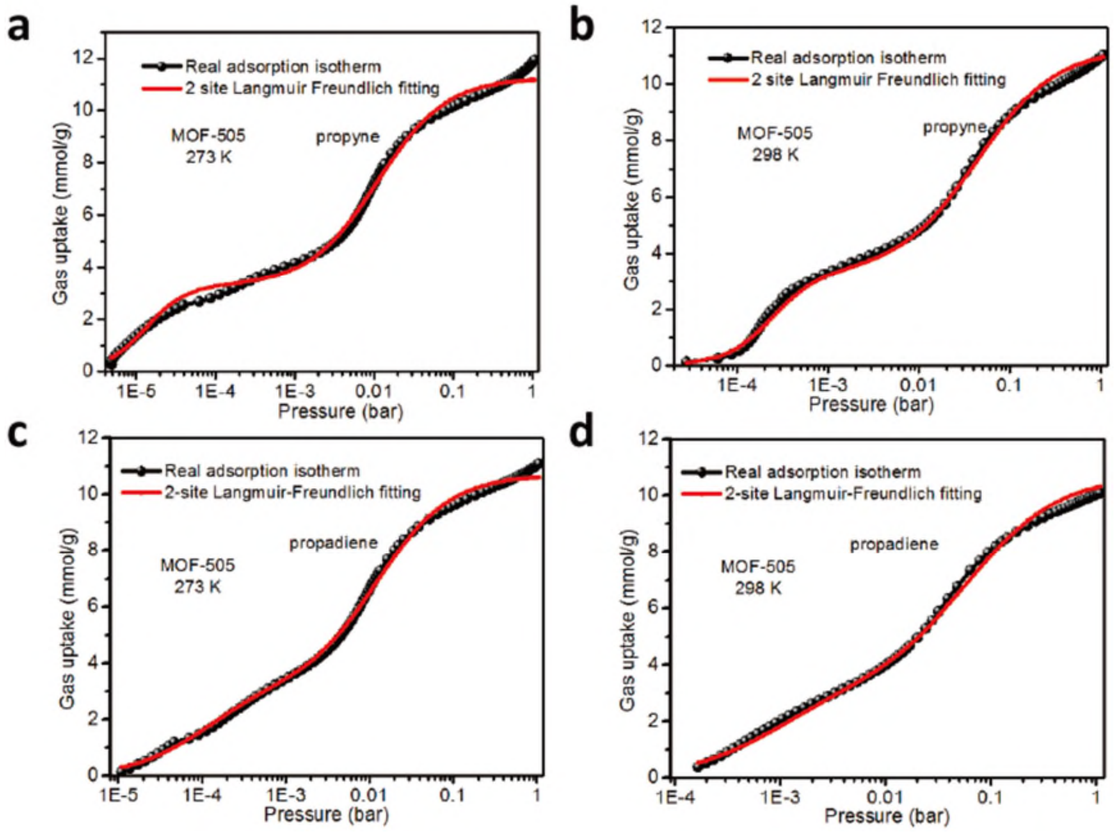

Supplementary Fig. 13 Dual-site Langmuir-Freundlich model fits of propyne and propadiene adsorption isotherms at 273 K and 298 K in MOF-505. **a, b** propyne. **c, d** propadiene.

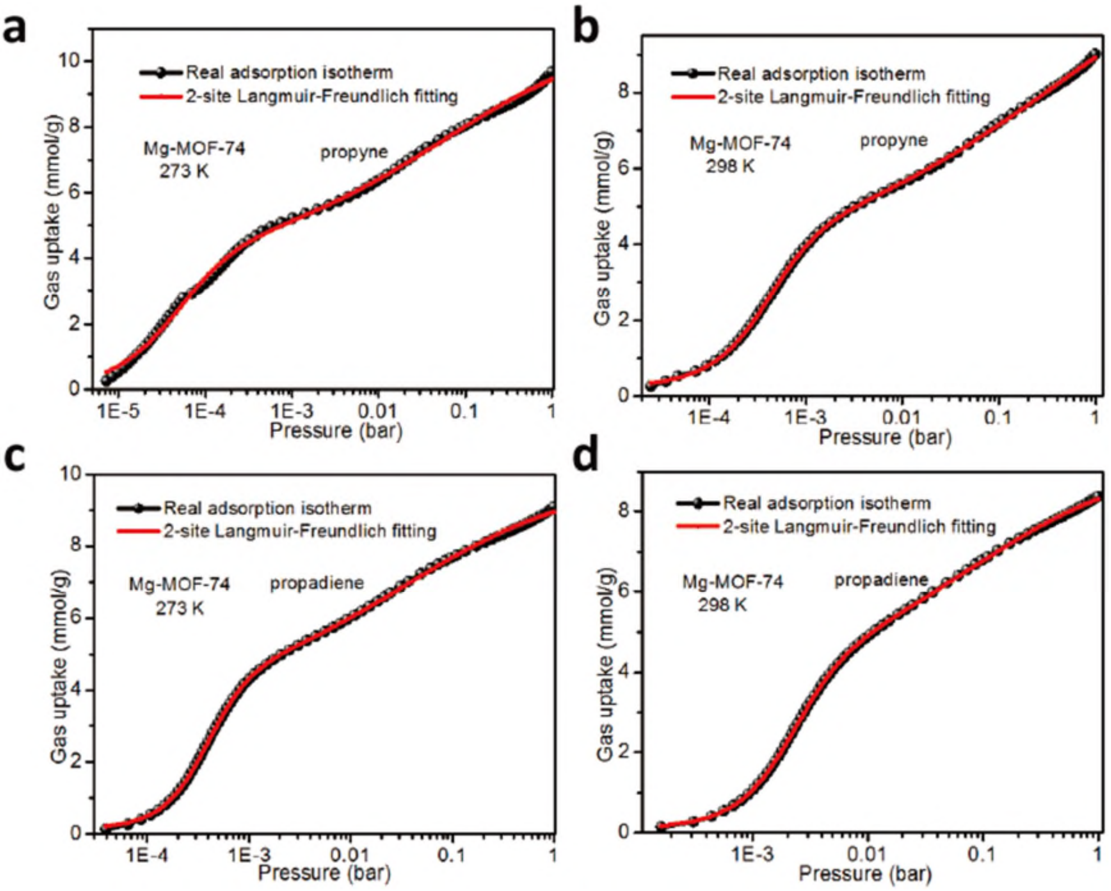

Supplementary Fig. 14 Dual-site Langmuir-Freundlich model fits of propyne and propadiene adsorption isotherms at 273 K and 298 K in Mg-MOF-74. **a, b** propyne. **c, d** propadiene.

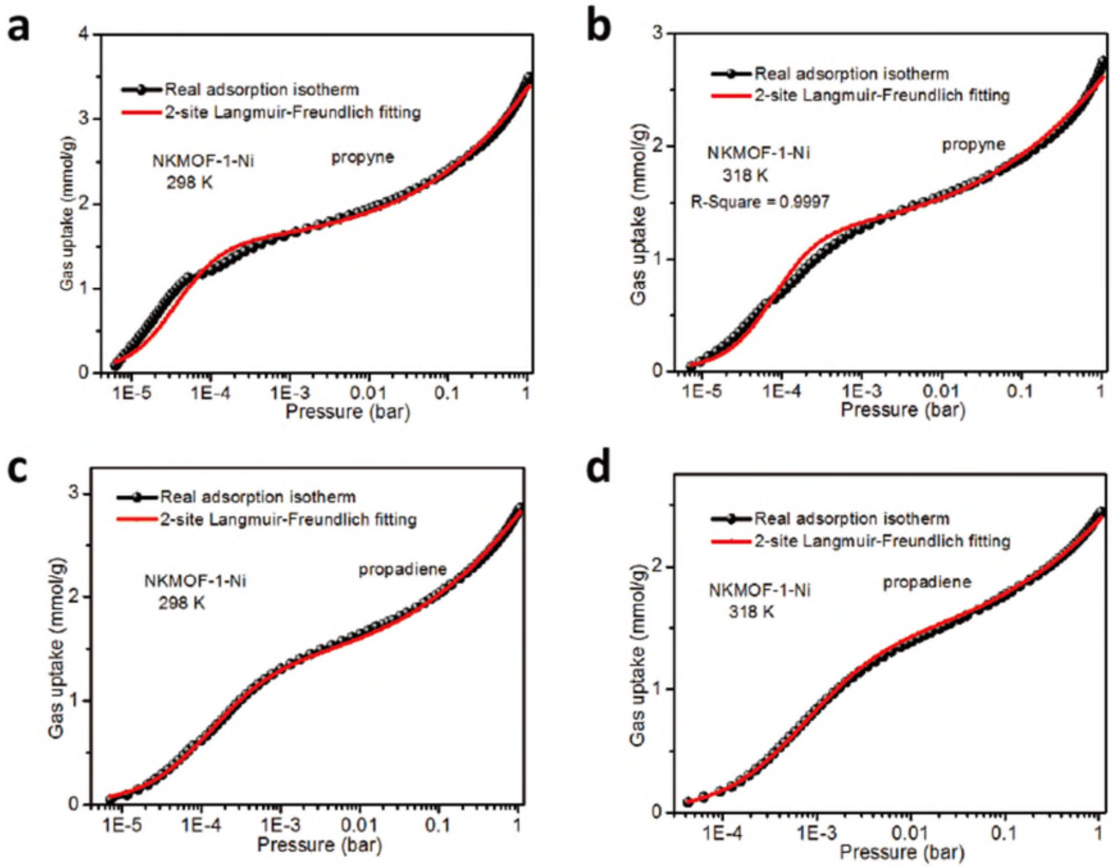

Supplementary Fig. 15 Dual-site Langmuir-Freundlich model fits of propyne and propadiene adsorption isotherms at 273 K and 298 K in NKMOF-1-Ni. **a, b** propyne. **c, d** propadiene.

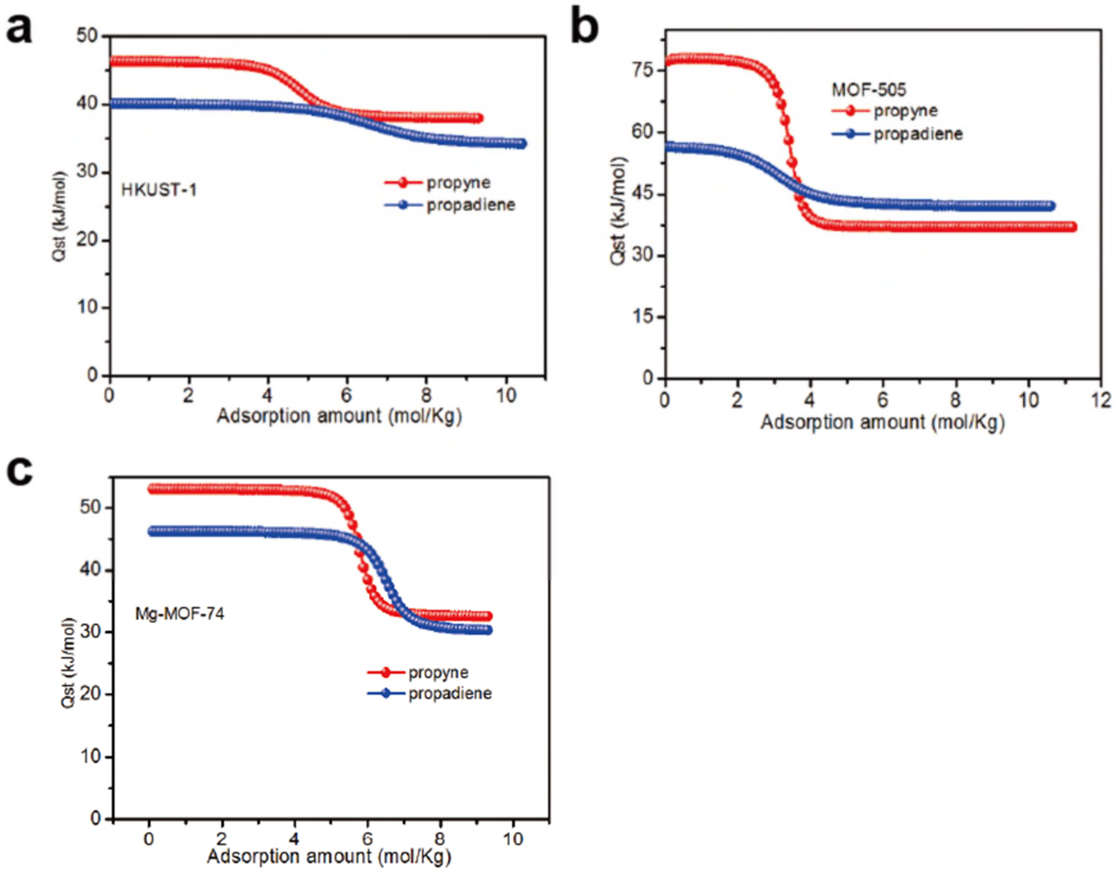

Supplementary Fig. 16 The adsorption isosteric enthalpy (Q_{st}) of propyne and propadiene in (**a**) HKUST-1, (**b**) MOF-505 and (**c**) Mg-MOF-74.

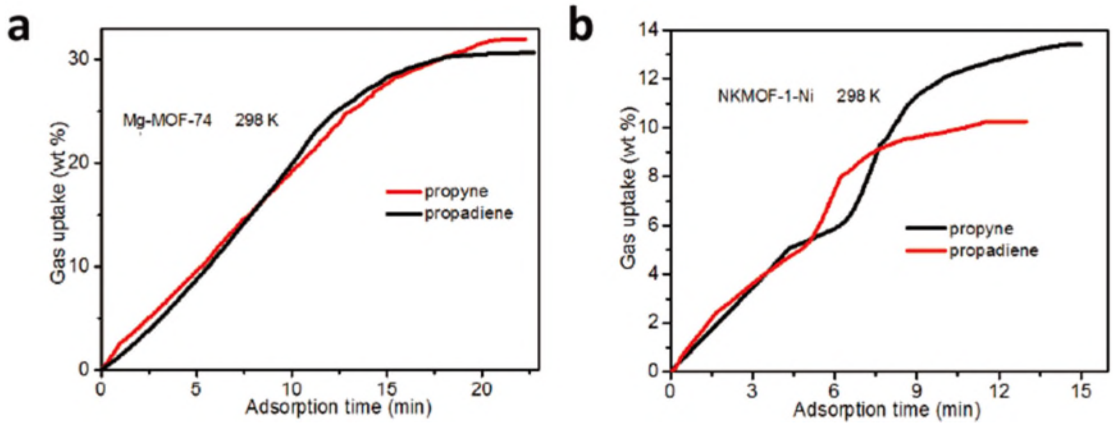

Supplementary Fig. 17 Gravimetric propyne and propadiene sorption kinetics at 298 K. **a** Mg-MOF-74. **b** NKMOF-1-Ni.

Supplementary Table 1. The BET surface, pore size and crystal density of the selected MOFs.

MOF Types	MOFs name	BET surface (m^2/g)	Pore size (Å)	Crystal density (g/cm^3)	Ref
MOFs with open metal sites	HKUST-1	1850	7-10	0.879	(*1*)
	MOF-505	1830	8.3-10.1	0.992	(*2*)
	Mg-MOF-74	1415	11	0.920	(*3*)
	NKMOF-1-Ni	420	5.7	1.12	(*4*)
	MIL-100(Cr)	3100	29	0.784	(*5*)
	MIL-100 (Fe)	2800	32	1.064	(*6*)
	MIL-101(Cr)	4100	29-34	--	(*7*)
MOFs with strong binding sites	SIFSIX-2-Cu-i	735	4.4	1.247	(*8*)
	SIFSIX-3-Ni	250	4.2	1.570	(*9*)
	UTSA-200	612	3.4	1.417	(*10*)
	ZU-62	476	3.2	1.378	(*11*)
MOFs without strong binding sites	UIO-66	1390	6	1.19	(*12*)
	UIO-67	1680	9	1.002	(*12*)
	ZIF-8	1630	3.5	1.067	(*13*)

Supplementary Table 2. Dual-site Langmuir parameter fits for propyne and propadiene in **HKUST-1** at 273 K and 298 K.

Guest	Temperature (K)	Site A		Site B	
		$q_{A,sat}$ (mol Kg^{-1})	B_a (Pa^{-1})	$q_{B,sat}$ (mol Kg^{-1})	b_B (Pa^{-1})
propyne	273	4.8	8.999E-02	6.2	4.727E-04
	298	4.8	1.620E-02	6.2	1.166E-04
propadiene	273	6.5	6.160E-03	4	1.461E-04
	298	6.5	1.394E-03	4	4.165E-05

Supplementary Table 3. Dual-site Langmuir parameter fits for propyne and propadiene in **Mg-MOF-74** at 273 K and 298 K.

Guest	Temperature (K)	Site A		Site B	
		$q_{A,sat}$ (mol Kg^{-1})	b_A (Pa^{-1})	$q_{B,sat}$ (mol Kg^{-1})	b_B (Pa^{-1})
propyne	273	5.8	1.307E-01	3.6	1.865E-04
	298	5.8	1.837E-02	3.6	5.617E-05
propadiene	273	6.5	1.493E-02	2.9	6.081E-05
	298	6.5	2.697E-03	2.9	1.999E-05

Supplementary Table 4. Dual-site Langmuir-Freundlich parameter fits for propyne and propadiene in **MOF-505** at 273 K and 298 K.

Guest	Temperature (K)	Site A			Site B		
		$q_{A,sat}$ (mol Kg^{-1})	b_A (Pa^{-vA})	V_A	$q_{B,sat}$ (mol Kg^{-1})	b_B (Pa^{-vB})	V_B
propyne	273	3.3	6.348E-01	1.7	7.95	9.097E-04	1
	298	3.3	4.484E-03	1.7	7.95	2.317E-04	1
propadiene	273	7.5	7.954E-04	1	3.2	9.413E-02	1
	298	7.5	1.684E-04	1	3.2	1.145E-02	1

Supplementary Table 5. Dual-site Langmuir-Freundlich parameter fits for propyne and propadiene in **NKMOF-1-Ni** at 298 K and 318 K.

Guest	Temperature (K)	Site A			Site B		
		$q_{A,sat}$ (mol Kg^{-1})	b_A (Pa^{-vA})	V_A	$q_{B,sat}$ (mol Kg^{-1})	b_B (Pa^{-vB})	V_B
propyne	298	1.5	1.510E-01	1.48	9	3.651E-03	0.3
	318	1.2	4.788E-02	1.48	4.4	6.526E-03	0.3
propadiene	298	1.35	7.612E-02	1	3.85	3.377E-03	0.4
	318	1.35	1.355E-02	1	3.85	2.032E-03	0.4

Supplementary Table 6: Calculated averaged total potential energies (in kJ mol^{-1}) for a single propyne and propadiene molecule, individually, positioned about the open-metal sites in **HKUST-1**, **MOF-505**, and **Mg-MOF-74** and within the tetrahedral cage in **HKUST-1** as determined from CMC simulations at 298 K/0.10 atm.

MOF	Adsorbate	MOF-Adsorbate Energy (kJ mol^{-1})
**HKUST-1**	Propyne (Open-Metal Site)	– 54.15
	Propadiene (Open-Metal Site)	– 50.74
	Propyne (Tetrahedral Cage)	– 47.37
	Propadiene (Tetrahedral Cage)	– 48.85
**MOF-505**	Propyne	– 89.17
	Propadiene	– 43.19
**Mg-MOF-74**	Propyne	– 78.46
	Propadiene	– 60.62

Supplementary References

1. Lin, Kuen-Song, *et al*. Synthesis and characterization of porous HKUST-1 metal organic frameworks for hydrogen storage. *Int. J. Hydrogen. Energy.* **37,** 13865-13871 (2012).

2. Chen, B. *et al.* High H_2 adsorption in a microporous metal-organic framework with open metal sites. *Angew. Chem., Int. Ed.* **44**, 4745-4749 (2005).

3. Caskey, S. R., Wong-Foy, A. G. & Matzger, A. J. Dramatic tuning of carbon dioxide uptake via metal substitution in a coordination polymer with cylindrical pores. *J. Am. Chem. Soc*. **130**, 10870-10871 (2008).

4. Peng, Y. L. *et al.* A robust heterometallic ultramicroporous MOF with ultrahigh selectivity for propyne/propylene separation. *J. Mater. Chem. A*, **9**, 2850-2856 (2021).

5. Férey, G. *et al.* A hybrid solid with giant pores prepared by a combination of targeted chemistry, simulation, and powder diffraction. *Angew. Chem., Int. Ed.* **116**, 6456-6461(2004).

6. Horcajada, P. *et al.* Synthesis and catalytic properties of MIL-100 (Fe), an iron (III) carboxylate with large pores. *Chem. Commun.* **27**, 2820-2822 (2007).

7. Férey, G. *et al.* A chromium terephthalate-based solid with unusually large pore volumes and surface area. *Science* **309**, 2040-2042 (2005).

8. Nugent, P. *et al.* Porous materials with optimal adsorption thermodynamics and kinetics for CO_2 separation. *Nature* **495**, 80-84 (2013).

9. Kumar, A. *et al.* Direct air capture of CO_2 by physisorbent materials. *Angew. Chem.*,

Int. Ed. **54**, 14372-14377 (2015).

10. Li, B. *et al.* An ideal molecular sieve for acetylene removal from ethylene with record selectivity and productivity. *Adv. Mater.* **29**, 1704210 (2017).

11. Yang, L. *et al.* An asymmetric anion-pillared Metal-organic framework as a multisite adsorbent enables simultaneous removal of propyne and propadiene from propylene. *Angew. Chem., Int. Ed.* **130**, 13329-13333 (2018).

12. Cavka, J. H. *et al.* A new zirconium inorganic building brick forming metal organic frameworks with exceptional stability. *J. Am. Chem. Soc.* **130**, 13850-13851 (2008).

13. Park, K. S. *et al.* Exceptional chemical and thermal stability of zeolitic imidazolate frameworks. *Proc. Natl. Acad. Sci.* **103**, 10186-10191 (2006).

14. Krishna, R. The maxwell-stefan description of mixture diffusion in nanoporous crystalline materials. *Micropor. mesopo. mat.* **185**, 30-50 (2014).

15. Krishna, R. Methodologies for evaluation of Metal－organic frameworks in separation applications. *RSC Adv.* **5**, 52269-52295 (2015).

15. Krishna, R. Screening metal-organic frameworks for mixture separations in fixed-bed adsorbers using a combined selectivity/capacity metric. *RSC Adv.* **7**, 35724-35737 (2017).

16. Krishna, R. Methodologies for screening and selection of crystalline microporous materials in mixture separations. *Sep. Purif. Technol.* **194**, 281-300 (2018).

17. Krishna, R. Metrics for evaluation and screening of metal-organic frameworks for applications in mixture separations. ACS Omega 5, 16987-17004 (2020).

18. Chui, S. S. Y., Lo, S. M. F., Charmant, J. P., Orpen, A. G. & Williams, I. D. A chemically functionalizable nanoporous material $[Cu_3(TMA)_2(H_2O)_3]$ n. *Science* **283**, 1148-1150 (1999).

19. Chen, B. *et al.* High H_2 adsorption in a microporous metal-organic framework with open metal sites. *Angew. Chem. Int. Ed.* **44**, 4745-4749 (2005).

20. Pham, T. *et al.* Understanding the H_2 sorption trends in the M-MOF-74 series (M= Mg, Ni, Co, Zn). *J. Phys. Chem. C* **119**, 1078-1090 (2015).

21. Lennard-Jones, J. E. On the determination of molecular fields. II. From the equation of state of gas. *Proc. Roy. Soc. A* **106**, 463-477 (1924).

22. Rappé, A. K. *et al.* UFF, a full periodic table force field for molecular mechanics and molecular dynamics simulations. *J. Am. Chem. Soc.* **114**, 10024-10035 (1992).

23. Valiev, M. *et al.* NWChem: A comprehensive and scalable open-source solution for large scale molecular simulations. *Comput. Phys. Commun.* **181**, 1477-1489 (2010).

24. Cornell, W. D. *et al.* A second generation force field for the simulation of proteins, nucleic acids, and organic molecules *J. Am. Chem. Soc.* **117**, 5179-5197 (1995).

25. Stevens,W. J., Basch, H. & Krauss, M. Compact effective potentials and efficient shared-exponent basis sets for the first-and second-row atoms. *J. Chem. Phys.* **81**, 6026-6033 (1984).

26. Hay, P. J., Wadt, W. R. Ab initio effective core potentials for molecular calculations. Potentials for the transition metal atoms Sc to Hg. *J. Chem. Phys.* **82**, 270-283 (1985).

27. LaJohn, L. A., Christiansen, P. A., Ross, R. B., Atashroo, T. & Ermler, W. C. A binitio relativistic effective potentials with spin-orbit operators. III. Rb through Xe. *J. Chem. Phys.* **87**, 2812-2824 (1987).

28. Duijnen, P. T. V. & Swart, M. Molecular and atomic polarizabilities: Thole's model revisited. *J. Phys. Chem. A* **102**, 2399-2407 (1998).

29. Forrest, K. A. *et al.* Simulation of the mechanism of gas sorption in a Metal－organic framework with open metal sites: Molecular hydrogen in PCN-61. *J. Phys. Chem. C* **116**, 15538-15549 (2012).

30. Peng,Y.-L. *et al.* Robust microporous metal-organic frameworks for highly efficient and simultaneous removal of propyne and propadiene from propylene *Angew. Chem. Int. Ed.* **58**, 10209-10214 (2019).

31. Ewald, P. P. Die Berechnung optischer und elektrostatischer Gitterpotentiale. *Ann. Phys.* **369**, 253-287(1921).

32. Wells, B. A. & Chaffee, A. L. Ewald summation for molecular simulations. *J. Chem. Theory Comput.* **11**, 3684-3695 (2015).

33. Applequist, J., CarlK. J. R. & Fung, K. Atom dipole interaction model for molecular polarizability. Application to polyatomic molecules and determination of atom polarizabilities. *J. Am. Chem. Soc*. **94**, 2952-2960 (1972).

34. Thole, B. T. Molecular polarizabilities calculated with a modified dipole interaction. *Chem. Phys.* 59, 341-350 (1981).

35. Bode,K. A. & Applequist, J. A new optimization of atom polarizabilities in halomethanes, aldehydes, ketones, and amides by way of the atom dipole

interaction model. *J. Phys. Chem.* **100**, 17820-17824 (1996).

36. McLaughlin, K., Cioce, C. R., Pham, T., Belof, J. L. & Space, B. Efficient calculation of many-body induced electrostatics in molecular systems. *J Chem Phys* 139, 184112 (2013).

37. Belof, J. L. & Space, B. Massively Parallel Monte Carlo (MPMC). Available on GitHub. https://github.com/mpmccode/mpmc (2012).

38. Franz, D. M. *et al.* MPMC and MCMD: Free High-Performance Simulation Software for Atomistic Systems. *Adv. Theory Simul.* **2**, 1900113 (2019).

39. Kirkpatrick, S., Gelatt, C. D., Vecchi, M. P. Optimization by simulated annealing. *Science* **220**, 671-680 (1983).

40. Frenkel, D., Smit, B. (2002). *Understanding Molecular Simulation: From Algorithms to Applications. Chapter 5* (New York, p112 – p114, 2002)
